# *Phaeocystis globosa* and diatom blooms promote distinct bacterial communities and associations in a coastal ecosystem

**DOI:** 10.1111/1758-2229.13313

**Published:** 2024-07-10

**Authors:** Dimitra‐Ioli Skouroliakou, Elsa Breton, Urania Christaki

**Affiliations:** ^1^ UMR CNRS 8187 LOG, Université Littoral Côte d’Opale, Université de Lille Wimereux France; ^2^ Present address: Laboratory of Protistology and Aquatic Ecology, Department of Biology Ghent University Ghent Belgium

## Abstract

Phytoplankton and bacteria form the foundation of marine food webs. While most studies on phytoplankton bloom influence on bacteria dynamics focus on diatom‐dominated blooms due to their global ecological significance, it is unclear if similar patterns extend to other species that compete with diatoms like *Phaeocystis* spp. This study aimed to contribute to the understanding of associations between phytoplankton and bacteria in a temperate ecosystem. For this, we studied the dynamics of phytoplankton and bacteria, combining 16S metabarcoding, microscopy, and flow cytometry over 4 years (282 samples). Phytoplankton and bacterial communities were studied throughout the year, particularly during contrasting phytoplankton blooms dominated by the Haptophyte *Phaeocystis globosa* or diatoms. We applied extended local similarity analysis (eLSA) to construct networks during blooming and non‐blooming periods. Overall, the importance of seasonal and species‐specific interactions between phytoplankton and bacteria is highlighted. In winter, mixed diatom communities were interconnected with bacteria, indicating a synergistic degradation of diverse phytoplankton‐derived substrates. In spring, despite the intensity variations of *P. globosa* blooms, the composition of bacterial communities remained consistent over several years, suggesting establishing a stable‐state environment for bacterial communities. Specific associations between monospecific diatom blooms and bacteria were evidenced in summer.

## Graphical abstract

Bacteria and phytoplankton succession and their putative interactions were investigated in a coastal ecosystem across multiple years. During winter, in the presence of a diverse diatom community, bacteria were associated with, and between, all phytoplankton. In contrast, during monospecific *Phaeocystis globosa* and diatom blooms, specific bacterial genera were strongly associated with the blooming species; and promoted distinct bacterial communities.

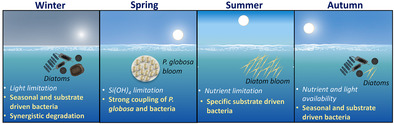

## INTRODUCTION

Marine phytoplankton contributes up to 50% of global primary production (Falkowski et al., 1998; Field et al., 1998), while bacteria in the marine environment are responsible for remineralizing at least half of this production (Azam et al., 1983; Worden et al., 2015). The interactions between bacteria and phytoplankton represent a fundamental ecological relationship in marine ecosystems, spanning from cooperative to competitive (reviewed in Seymour et al., 2017). For example, a typically cooperative (i.e., mutualistic) relationship is the release of dissolved organic carbon by phytoplankton, which provides carbon and energy to bacteria. In return, bacteria are the key engineers in the transformation of nutrients and trace elements to support phytoplankton growth (e.g., Durham et al., 2015). Thus, bacteria and phytoplankton interactions are a determinant force modulating microbial community structure (e.g., reviewed in Fuhrman et al., 2015; Genitsaris et al., 2015; Lima‐Mendez et al., 2015; Steinrücken et al., 2023), biomass production, carbon and oxygen fluxes (Azam & Malfatti, 2007; Cole, 1982). On the other hand, algicidal bacteria induce negative effects on phytoplankton targets (e.g., Mayali & Azam, 2004; Meyer et al., 2017). The associations between phytoplankton and bacteria explored through in situ and experimental studies evidenced recurrent bacteria seasonal patterns at taxonomic and functional levels (e.g., Bunse & Pinhassi, 2017; Teeling et al., 2012, 2016; Ward et al., 2017) and specific preferences between the substrate produced by phytoplankton blooms and several bacteria (Buchan et al., 2014; Delmont et al., 2014; Dlugosch et al., 2023; Landa et al., 2016).

Processing phytoplankton‐derived organic matter requires different adaptive strategies by bacteria (Buchan et al., 2014; Koch, 2001; Lauro et al., 2009). For example, ‘oligotrophs’ grow in low organic matter environments, such as in winter conditions and deep offshore waters, while ‘copiotrophs’ develop during high productivity periods, such as phytoplankton blooms (e.g., Giovannoni et al., 2014; Lemonnier et al., 2020). Bacteria also present different degrees of ecological specialization, with ‘generalists’ being able to assimilate a broad range of substrates and ‘specialists’ assimilating a narrower range of substrates (e.g., Mou et al., 2008). Furthermore, abiotic factors including temperature, water column characteristics and nutrients impact bacterial succession (e.g., Bunse & Pinhassi, 2017).

Because of the ecological significance of diatoms at a global scale (Tréguer et al., 2018), most studies on the influence of phytoplankton blooms on bacteria dynamics are related to diatom‐dominated blooms (e.g., Arandia‐Gorostidi et al., 2022; Chafee et al., 2018; Schäfer et al., 2002; Teeling et al., 2012, 2016). It remains unclear whether similar patterns are extended to other phytoplankton, particularly those that may compete with diatoms, such as *Phaeocystis* spp., a cosmopolitan Haptophyte that often dominates spring blooms in coastal waters (e.g., Peperzak et al., 1998; review in Schoemann et al., 2005). The availability of distinct polysaccharides in the *Phaeocystis* spp. colony matrix and other chemical properties, such as amino‐sugars and abundant dimethylsulfoniopropionate (DMSP), promote a unique bacterial consortium in the *Phaeocystis* spp. microenvironment (Mars Brisbin et al., 2022; Shen et al., 2011; Solomon et al., 2003). The few existing in situ studies focused on *Phaeocystis* spp. and associated bacteria were conducted over short periods and showed shifts in bacterial community composition and activity at different phases of the bloom (Alderkamp et al., 2006; Gibson et al., 2022; Lamy et al., 2009).

The present study was conducted in the coastal waters of the meso‐eutrophic Eastern English Channel (EEC), dominated by blooms of two phylogenetically distant and competing groups, Haptophytes (i.e., *Phaeocystis globosa*) and Bacillariophyta (diatoms). Specifically, the phytoplankton succession in this area is characterized by a rich diatom winter community, recurrent *P. globosa* bloom in spring (e.g., Breton et al., 2006; Christaki et al., 2023), and transient diatom blooms in summer (Skouroliakou et al., 2022). We hypothesized that the bacterial seasonal community structure was associated with different seasonal phytoplankton communities but was also affected by *P. globosa* and transient summer diatom blooms. Based on this hypothesis the objectives were: (i) to describe bacterial seasonal patterns and taxon‐specific relationships with phytoplankton on a pluriannual scale; and (ii) to determine whether variable intensities of *P. globosa* blooms were accompanied by similar bacterial community structures and whether they were different from those of diatom blooms. The temporal patterns of phytoplankton and bacterial communities were investigated using microscopy, flow cytometry, and 16S rRNA gene amplicon sequencing at the surface coastal waters of the EEC from 2016 to 2020 (i.e., 282 samples). The extended local Similarity Analysis (eLSA) was used to investigate potential relations between phytoplankton and bacteria.

## EXPERIMENTAL PROCEDURES

### Sampling

Subsurface seawater samples were collected (2 m depth) at five neighbouring stations in the EEC (Figure S1). From 2016 to 2020, the SOMLIT (French Network of Coastal Observatories; https://www.somlit.fr/) coastal S1 and offshore S2 stations were sampled bi‐weekly. From 2018 to 2020, the sampling effort was intensified with weekly samplings at three additional stations (R1, R2, and R4) from the local monitoring transect ‘DYPHYRAD’ situated approximately 15 km north of the SOMLIT stations (Figure S1, Table S1). Higher frequency sampling (2–3 times per week) was also carried out after the end of the spring bloom in June–July and in autumn September–October at stations R1‐R4. The higher frequency was applied to catch rapidly changing bacteria and phytoplankton dynamics. The post‐spring bloom period (June–July) was chosen because high bacterial abundance and activity were observed after a *P. globosa* bloom (e.g., Lamy et al., 2009), as well as high diatom abundance and biomass (e.g., Breton et al., 2017). High‐frequency sampling was carried out in September and October because it is a transitional period from summer to winter conditions, and sporadic low‐intensity blooms may occur (e.g., Breton, 2000). Only three samples were collected in August due to the boat's unavailability during this month. For this reason, the August data are presented but not further discussed.

### Environmental variables

Sea surface temperature (T,°C) and salinity (S, PSU) were measured in situ with a conductivity‐temperature‐depth profiling system (CTD Seabird profiler SBE 25). The average subsurface daily PAR (Photosynthetic Active Radiation) experienced by phytoplankton in the water column for 6 days before sampling was obtained using global solar radiation (GSR, Wh m^−2^) recorded by the Copernicus Atmosphere Monitoring Service (CAMS) radiation service (http://www.soda-pro.com/web-services/radiation/cams-radiation-service). GSR was converted into PAR by assuming PAR to be 50% of GSR and by considering 1 W m^−2^ = 0.36 E m^−2^ d^−1^ (Morel & Smith, 1974). Seawater macronutrient concentrations, nitrate (NO_3_^−^), nitrite (NO_2_^−^), phosphate (PO_4_^3^‐), and silicate (Si(OH)_4_) were analysed according to Aminot and Kérouel (2004). Chlorophyll‐a (Chl‐*a*) concentration was measured by fluorometry (Lorenzen, 1966). Additional details on environmental data acquisition and analysis can be found at https://www.somlit.fr/en/.

### Phytoplankton

For diatoms and *P. globosa* (colonies, free flagellate, and colonial cells) counting, 110 mL water samples were collected and fixed with Lugol's‐glutaraldehyde solution (1% v/v, which does not disrupt *P. globosa*'s colonies (Breton et al., 2006)). Phytoplankton species were identified to the genus or species level when possible, using an inverted microscope (Nikon Eclipse TE2000‐S) at 100–400× magnification after sedimentation for 24 h in a 10 mL Hydrobios chamber (e.g., Breton et al., 2021). The bloom periods were defined based on abundance data according to the IFREMER REPHY/REPHYTOX thresholds based on 30‐year data at the French coasts, including those of the EEC (the REPHY/REPHYTOX station is situated 2 km west of the R1 station; https://www.phytobs.fr/Stations/Boulogne). These thresholds were ≥10^5^ or ≥10^6^ for diatoms (depending on the species) and ≥10^6^ for *P. globosa* (Belin et al., 2021).

### Bacteria

Seawater samples (1.5 mL) were fixed with paraformaldehyde (PFA) at a final concentration of 1%, then stored at 4°C for 40 min, flash‐frozen in liquid nitrogen, and then kept at −80°C until analysis. Bacteria were stained with SYBR Green I (Marie et al., 1999) and enumerated by flow cytometry with a CytoFlex cytometer (Beckman Coulter). Autotrophic bacteria (here mainly *Synechococcus*) were discriminated from non‐containing photosynthetic pigments bacteria based on their side scatter and red autofluorescence (Marie et al., 1999).

For bacterial diversity, four to seven litres of seawater, depending on the quantity of the particulate material in the water (i.e., until signs of clogging of the filter), were filtered onto 0.2 μm polyethersulfone (PES) membranes (142 mm, Millipore, U.S.A.) after pre‐screening through 150 μm nylon mesh (Millipore, U.S.A.) to remove metazoans. Filters were stored at −80°C until DNA extraction. A quarter of the PES filter was used for DNA extraction with the DNAeasy PowerSoil Pro kit (Qiagen, Germany). Then, the 16S rRNA gene V3‐V4 region was amplified with the bacterial primer pair 341F (CCTACGGGNGGCWGCAG) and 785R (GACTACHVGGGTATCTAATCC) (Klindworth et al., 2013). Pooled purified amplicons were then paired‐end sequenced on an Illumina MiSeq 2 × 300 platform (Genewiz South Plainfield, NJ, USA, approximate sequencing depth: 50 K reads/sample).

Paired‐end sequences were imported and demultiplexed in Qiime (Caporaso et al., 2012) based on each sample's 10 bp molecular identifier with the functions extract_barcodes.py (Qiime1) and demux emp‐paired (Qiime 2‐2018.8). Demultiplexed sequences without primers and barcodes were further processed in R‐software (R Core Team, 2021) using the ‘DADA2’ package v.1.20.0 (Callahan et al., 2016) to define amplicon sequence variants (ASVs). Sequences were quality‐filtered and trimmed, and chimeric sequences were removed before inferring ASVs with the ‘DADA2’ algorithm. Taxonomy was assigned for each ASV to the best taxonomic level using the SILVA database (release 138.1; Quast et al., 2013). A total number of 34,508 ASVs were identified from 11,294,899 reads in 282 samples. ASVs assigned to mitochondria, chloroplasts, and autotrophic bacteria (i.e., Cyanobacteria and Chlorophlexi) were removed from the dataset (‘phyloseq’ package, McMurdie & Holmes, 2014). ASVs that were taxonomically unclassified at phylum level or were not assigned to bacterial lineages were excluded. To note, Archaea (202 ASVs; contributing to 7.5% of total reads) were removed because the primers used were insufficiently targeting this domain (Klindworth et al., 2013). It is also worth noting that two previous studies in the area detected very low contribution of Archaea in the prokaryotic community (i.e., 7% of DAPI stained cells, and 0.1% of the total number of sequences; Lamy et al., 2009; Genitsaris et al., 2016). The ASVs kept for downstream analysis will be referred to from now on as ‘bacteria’. Singletons were also excluded, and the dataset was rarefied at the lowest number of reads (8000). Out of a total of 282 samples, 26 were excluded from the analysis with less than 8000 reads, resulting in 2,071,296 reads corresponding to 9745 ASVs in 256 samples. Raw sequencing data have been submitted to the Short Read Archive under BioProject number PRJNA917476. For more details on DNA barcoding and bioinformatic analysis, see Appendix S1.

### Statistical analyses

All data visualizations were performed in R version 4.1.0. (R Core Team, 2021) using the ‘ggplot2’ package (Wickham, 2016). The significance of each environmental variable in driving bacterial community structure was explored with the distance‐based RDA analysis using the ‘microeco’ package (Liu et al., 2021). Environmental variables (NO_3_^−^ + NO_2_^−^, PO_4_^3−^ Si(OH)_4_, T, S, and Chl‐*a*) were included in the db‐RDA and standardized before analysis with the ‘deconstand function’. Soft clustering was used to define synchronous dynamics in the 21 most abundant bacteria at the genus level of the whole data set, which comprised ≥1% number of total reads, with the ‘cluster’ package (Maechler et al., 2023). Alpha diversity indices (Richness, Shannon, Simpson (1‐D)) were calculated after rarefying the read depths based on the sample with the lowest reads count (i.e., 8000 reads) with the package ‘microeco’ (Liu et al., 2021). For more details on statistical analysis, see Appendix S1.

### Network analysis

Network analysis was performed on 38 samples associated with *P. globosa* and diatom blooms and 31 samples (from December to February) as a reference ‘non‐blooming’ period characterized by a mixed diatom community (total of 69 samples, Table S1). These samples were selected from the coastal S1 and R1 stations, which were most frequently sampled. The extended local similarity analysis (eLSA) was used to assess significant positive and negative correlations among (i) dominant bacteria at genus taxonomic level (i.e., ≥ 1% number of reads in the 69 selected samples), (ii) environmental variables including nutrients (NO_2_, NO_3_, PO_4_, Si(OH)_4_), PAR, temperature, salinity, and Chl‐*a* and (iii) dominant phytoplankton taxa at genus or species level presenting ≥10% of the biomass in the selected samples. Biomass for diatoms and *P. globosa* was assessed from biovolumes (Menden‐Deuer & Lessard, 2000; Schoemann et al., 2005; Table S2).

eLSA was chosen because it is optimized to detect non‐linear, time‐sensitive relationships that other network analyses cannot otherwise identify (Ruan et al., 2006; Xia et al., 2011). The eLSA analysis looks through time series networks for associations that are strong enough and likely to be realistic associations. eLSA calculates the highest local similarity score (LS) between any pair of factors considering the time series length (Ruan et al., 2006). Here, the analysis was conducted following the tutorial (https://bitbucket.org/charade/elsa/wiki/Manual), using a delay of one‐time point to identify time‐lag correlations, and without any delay (Ruan et al., 2006; Steele et al., 2015; Xia et al., 2011). Only correlations with *p* < 0.05 and false‐discovery rate corrected *q*‐value <0.05 (Storey & Tibshirani, 2003) were considered. Network visualization and statistics were performed with Cytoscape v3.9. (Shannon et al., 2003). The non‐parametric test Kruskal‐Wallis was applied to test how the network characteristics differed among subnetworks using the ‘PMCMRplus’ package in R (Pohlert, 2015). For more details on network analysis, see Appendix S1.

## RESULTS

### Environmental context

**FIGURE 1:**
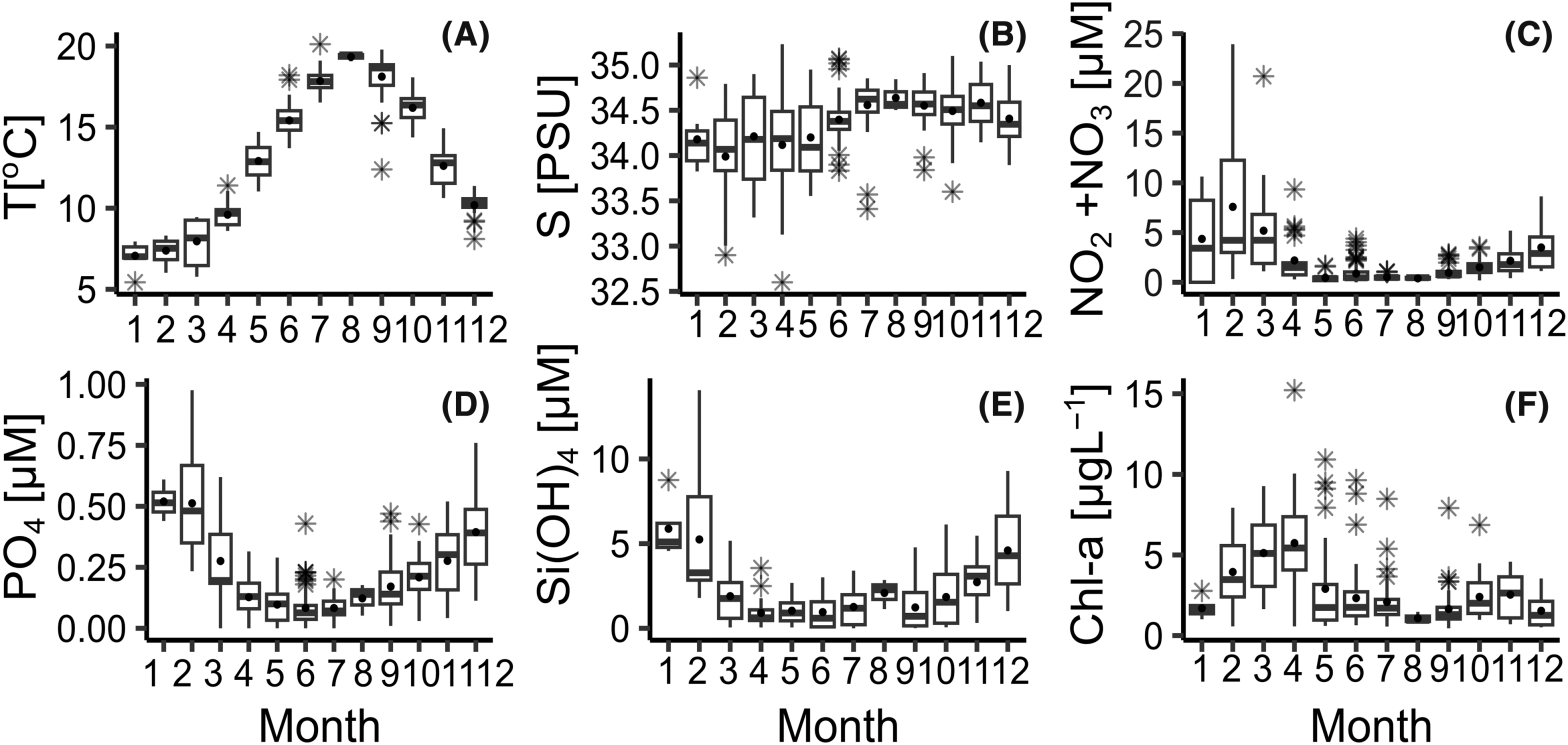
Seasonal variation of environmental variables: (A) Temperature [T,°C], (B) S: Salinity [S, PSU], (C) Nitrites and Nitrates [NO_2_ + NO_3_ μM], (D) Phosphates [PO_4_, μM], (E) Silicates: S [Si(OH)_4_, μM], (F) Chlorophyll‐a [Chl‐*a*, μg L^−1^] in the EEC at the DYPHYRAD and SOMLIT stations (Figure S1, Table S1) from March 2016 to October 2020.

The EEC's environmental parameters displayed seasonal patterns typical of temperate marine waters (Figure 1A–F, Table S3). Temperature increased from January to August and decreased from August to December, with mean monthly values ranging from 7.1 to 19.3^o^C (Figure 1A, Table S3). Mean monthly salinity ranged between 34.1 and 34.6 PSU, showing relatively high variability from winter to spring and several extreme values across seasons (Figure 1B, Table S3). Macronutrients decreased from January to March and increased from September to December (Figure 1C,D). The N/P ratio ranged from 0.4 to 316 (Table S3). Chl‐*a* values ranged from 0.5 to 15.2 μg L^−1^, with mean monthly values from 1.5 to 5.7 μg L^−1^. A comparison of the mean ranks (Kruskal Wallis and Nemenyi post‐hoc test) of environmental variables revealed significant differences in salinity, phosphate, silicate, and Chl‐*a* between stations (Figure S2, Table S3). However, the environmental variables were of the same range and showed the same seasonal variation at all stations (Figures 1 and S2A–F).

### Phytoplankton community structure and succession

**FIGURE 2:**
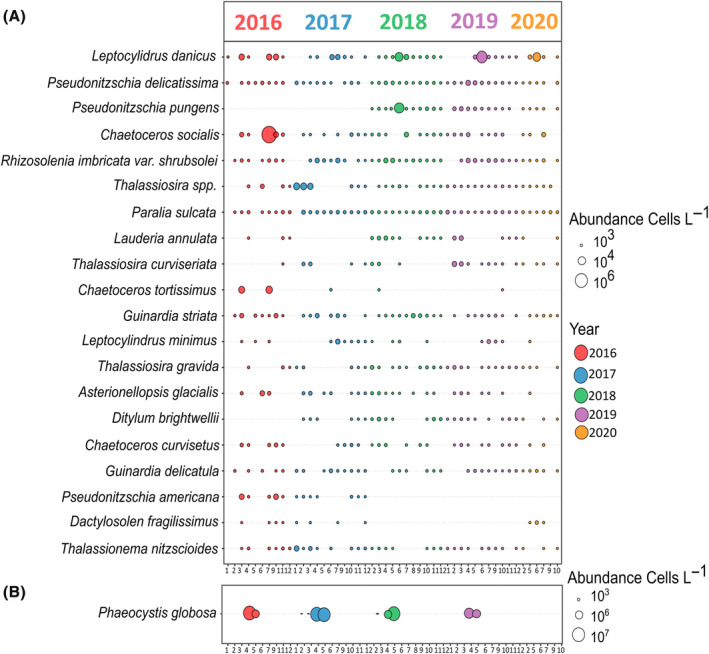
Bubble plot illustrating the mean monthly abundance (cells L^−1^) of (A) 20 most abundant diatoms and (B) *Phaeocystis globosa* occurring in the EEC at the DYPHYRAD and SOMLIT stations from March 2016 to October 2020. No data were available from April to May 2020 due to Covid‐19 restrictions. The size of the circles in the plot corresponds to the abundance classes (10^3^, 10^4^, and 10^6^). The colours correspond to the different years.

In this study, 90 diatoms were identified (83 at the species level and 7 at the genus level). Their abundance ranged from 4.0 × 10^3^ to 4.8 × 10^6^ cells L^−1^ (Figure S3). In winter, diatoms dominated phytoplankton with a mean abundance of 10^2^ × 10^3^ cells L^−1^. *Thalassiosira* spp. dominated winter phytoplankton in terms of abundance (24.7 ± 12.3 × 10^3^ cells L^−1^, 24%, Figure 2A) and biomass (3.2 ± 5.1 μgC L^−1^), accounting for 21% of diatom biomass. Besides *Thalassiosira* spp., several other diatom species contributed to the winter biomass (i.e., *Guinardia striata*; 21%, *Coscinodiscus* spp.; 16%, *Ditylum brightwellii*; 10%). Spring phytoplankton communities were dominated by *P. globosa*, which bloomed in April and May, featuring though differences in its abundance and biomass across years (i.e., annual maximum abundance ranged from 7.5 to 36.7 × 10^6^ cells L^−1^, Figures 2B and S3B, and annual maximum biomass from 132 to 1555 μg C L^−1^). The summer phytoplankton community showed several transient diatom blooms. *Chaetoceros socialis* showed a peak in July 2016, reaching 3.1 × 10^6^ cells L^−1^ (Figures 2A and S3A). The centric diatom *Leptocylindrus danicus* peaked in the summer from 2018 to 2020, with a maximum concentration of 5.9 × 10^6^ cells L^−1^, and the pennate diatom *Pseudo‐nitzschia pungens*, reached 4.8 × 10^6^ cells L^−1^ in June 2018 (Figures 2A and S3A). In autumn, phytoplankton was also dominated by diatoms; however, their abundance was two orders of magnitude lower than in summer (i.e., mean abundance of 3.9 × 10^4^ cells L^−1^).

Diatom communities showed high variability in alpha diversity (Figure S4). The diversity indices (Richness, Shannon, Simpson (1‐D)) showed an increasing trend from winter to spring, a decreasing trend from spring to summer, and an increasing trend from summer to autumn. Overall, the lowest values were observed during the *P. globosa* and diatom blooms (from April to June; Figure S4).

### Bacterial community structure and succession

According to metabarcoding data, the most abundant groups at the family taxonomic level were assigned to Actinomarinaceae (15.5%), followed by Flavobactericeae, contributing to 13.7% of the total number of reads. Actinomarinaceae exclusively consisted of the genus Candidatus *Actinomarina, the* most abundant genus across the dataset (15.5%). Flavobactericeae were more diversified, with the most dominant genera affiliated to NS5 marine group (2.8%), *Tenacibaculum* (2.5%), and NS4 marine group contributing to 2.1% in relative abundance. Bacterial richness mean monthly values were higher in autumn and winter and lower in spring and summer (Figure S5). The seasonality of mean monthly values was less pronounced for Simpson and Shannon indices (Figure S5). Overall, it is worth noting that comparing alpha diversity indices of phytoplankton to bacteria, the latter showed a less pronounced seasonality (Figures S4 and S5).

**FIGURE 3:**
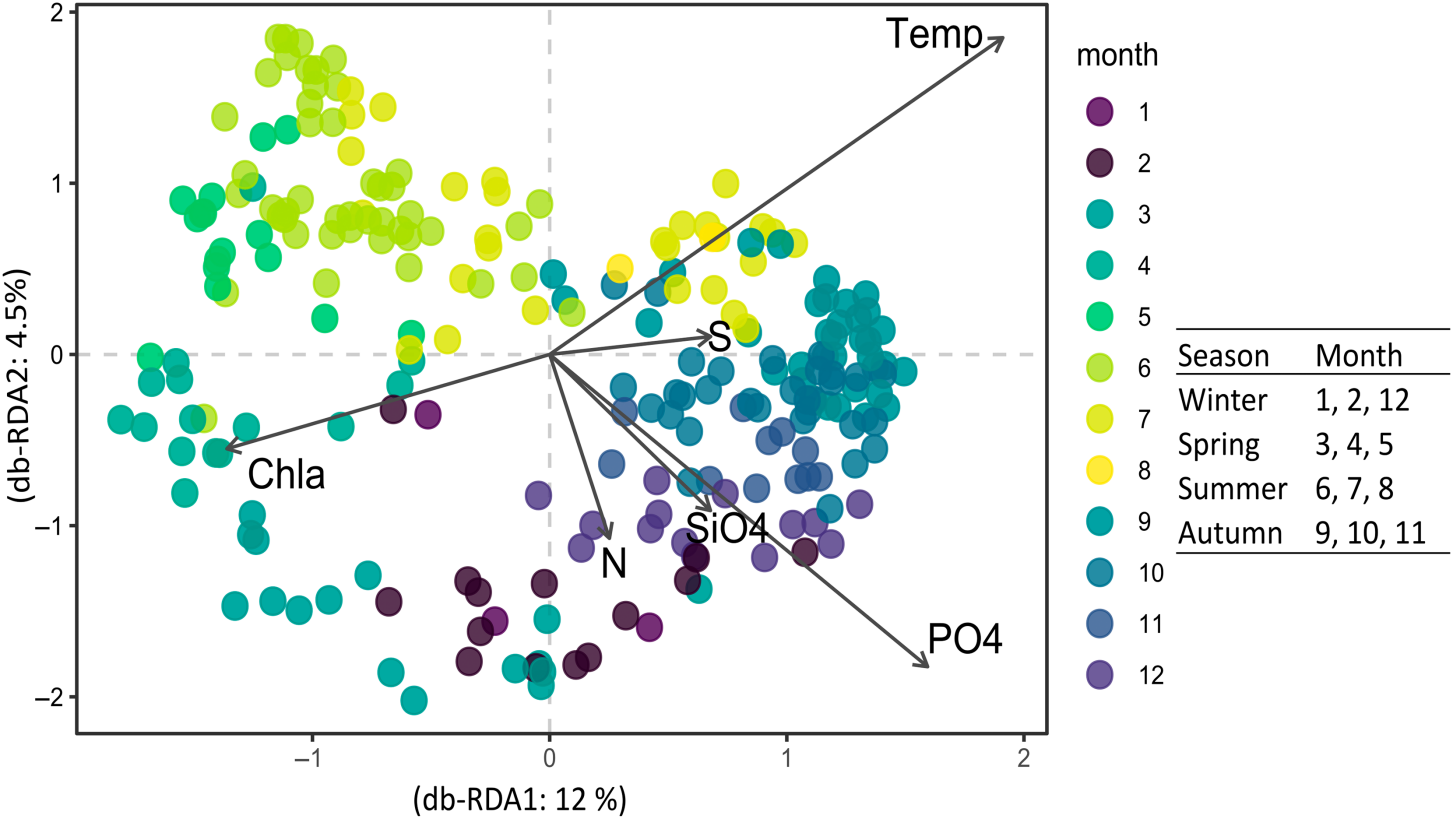
Distance‐based redundancy (db‐RDA) ordination illustrates the variations (i.e., 19.8% constrained variance; Tables S4, S5, S6) of bacterial communities based on metabarcoding data (samples are indicated with coloured dots) in relation to the environmental variables (black arrows) in the EEC at the DYPHYRAD and SOMLIT stations from March 2016 to October 2020.

The distance‐based redundancy analysis (db‐RDA) between environmental variables and bacterioplankton communities explained only 19.8% of the ‘constrained variance’ of the bacterial community composition (Figure 3; Table S4). The db‐RDA1 and db‐RDA2 contributed to 12% and 4.5% of the total variance (Figure 3; Table S5). Despite the low score of the constrained variance explained, bacterial communities showed a clear seasonal pattern on the db‐RDA plot (Figure 3). Autumn (from September to November) communities formed tighter groups on the db‐RDA plot, while summer ‐which presented diatom transient blooms‐ were more dispersed. Winter communities were associated to macronutrients (NO_2_ + NO_3_, PO_4_, Si(OH)_4_), spring communities to Chl‐*a*, and summer communities to temperature (Figure 3). The permutation ANOVA test showed that temperature, phosphates, and nitrites and nitrates significantly contributed to the overall variability (*p* < 0.001) respectively (Table S6).

**FIGURE 4:**
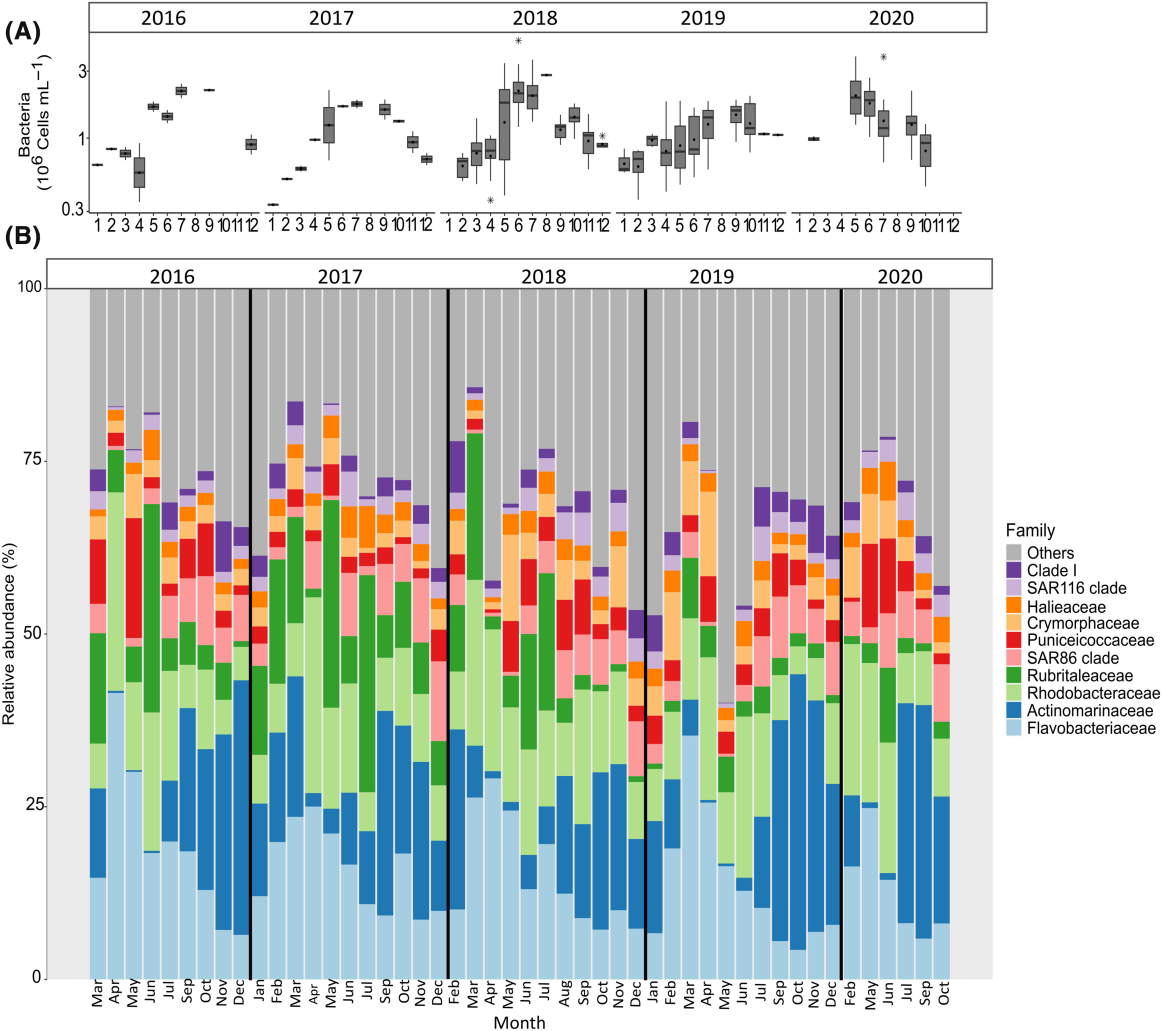
(A) Seasonal variation of mean monthly abundance (cells L^−1^) of bacteria enumerated by cytometry in the EEC at the SOMLIT and DYPHYRAD stations from March 2016 to October 2020, the Y axis is log10 transformed. (B) Seasonal variation of the 10 most abundant families based on rRNA gene metabarcoding data.

Non‐pigmented bacteria counted by flow cytometry abundance ranged from 0.3 to 4.9 × 10^6^ cells mL^−1^; their abundance increased from winter to summer and decreased from summer to autumn. Higher abundance was always evidenced after the wane of *P. globosa* bloom in June and July (Figure 4A). Among the 10 dominant bacterial families accounting for 69.8% of total read abundance, three families (Actinomarinaceae, Flavobacteriaceae, and Rhodobacteraceae) contributed to 41.2% in relative read abundance of the entire dataset (Figure 4B). Actinomarinaceae dominated bacteria community in winter (from December to February), and autumn (from September to November) reaching a maximum of 39% relative abundance of reads in winter (Figure 4B). However, during the spring (from March to April) and summer (from June to July) blooms, Flavobacteriaceae and Rhodobacteraceae relative abundance increased, dominating over Actinomarinaceae. Flavobacteriaceae ranged from 4% in winter to 40% in summer, respectively (Figure 4B).

**FIGURE 5:**
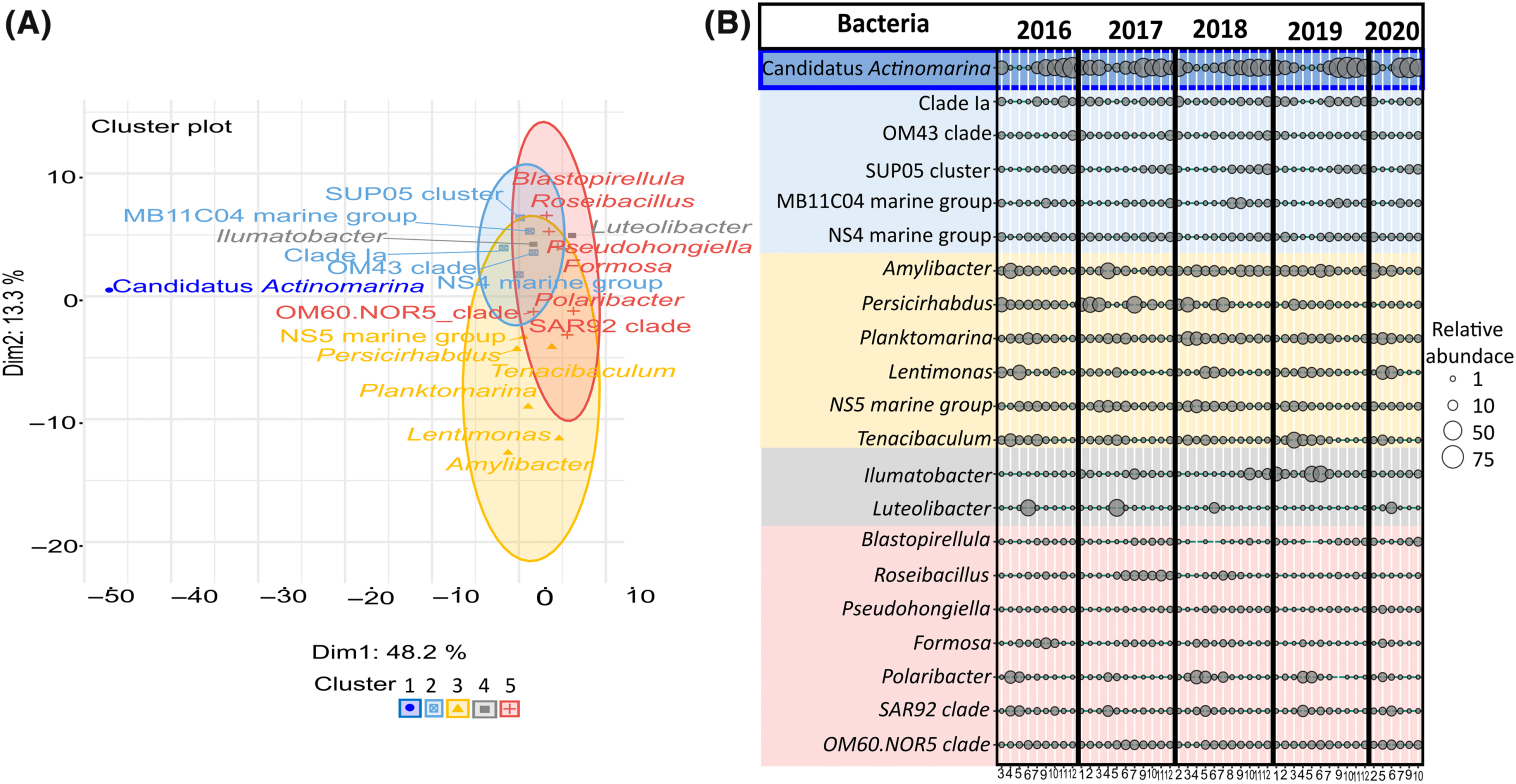
(A) Soft clustering of the 21 most abundant bacteria genera (i.e., ≥1% in read relative abundance in the entire dataset; 282 samples). The colours correspond to clusters defined by soft clustering analysis (cluster 1: Dark blue, cluster 2: Light blue, cluster 3: Yellow, cluster 4: Grey, cluster 5: Red) and the dots, squares, crosses, or triangles to bacteria (See also Table S7). (B) Bubble plot illustrating the mean relative abundance of 21 dominant bacteria genera (i.e., ≥1% reads relative abundance in the entire dataset; 282 samples). The size of the circles in the plot corresponds to the relative abundance classes (1%, 10%, 50%, and 75%). The colours correspond to clusters as assigned by soft clustering.

A total of 21 genera of the most abundant bacteria (i.e., ≥1% relative read abundance in the entire dataset; 282 samples) were assigned to five clusters based on their relative abundance (Figure 5A). All bacteria included in this analysis had a membership value of at least 0.7; thus, they predominantly belonged to one cluster, and no bacteria were equally assigned to multiple clusters (Table S7). Cluster 1 was solely composed of Candidatus *Actinomarina*, which was abundant in autumn and winter samples, contributing in more than 50% read relative abundance (Figure 5B). Cluster 2 was composed of six bacteria (i.e., Clade Ia, OM43 clade, SUP05 cluster, MB11C04 marine group, and NS4 marine group). These bacteria were less abundant than Candidatus *Actinomarina*, but they also peaked in autumn and winter (Figure 5B). Cluster 3 was composed of the following six bacteria: *Amylibacter*, *Persicirhabdus*, *Planktomarina*, *Lentimonas*, NS5 marine group, and *Tenacibaculum* (Figure 5B). The same six bacteria genera were dominant in spring during *P. globosa* blooms across years, despite the differences in bloom intensity (Figures 5B and 2B). Cluster 4 was composed of two bacteria belonging to *Ilumatobacter* and *Luteolibacter* genera, which were abundant in summer during the diatom blooms (Figure 2A). *Ilumatobacter* showed maximum relative abundance in June (i.e., 32%) during the *L. danicus* bloom in 2019. *Luteolibacter* showed peaks in June 2016 (i.e., 33.5%), in 2018 (i.e., 25%), and 2020 (i.e., 54%; Figure 5B). Cluster 5 was composed of seven bacteria (i.e., *Blastopirellula*, *Roseibacillus*, *Pseudohongiella*, *Formosa*, *Polaribacter*, SAR92 clade, OM60NOR5 clade) showing fluctuations (e.g., *Polaribacter*, *Roseibacillus*), or stable contribution in read relative abundance across years (Figure 5B).

### Bacteria putative associations with phytoplankton

**FIGURE 6:**
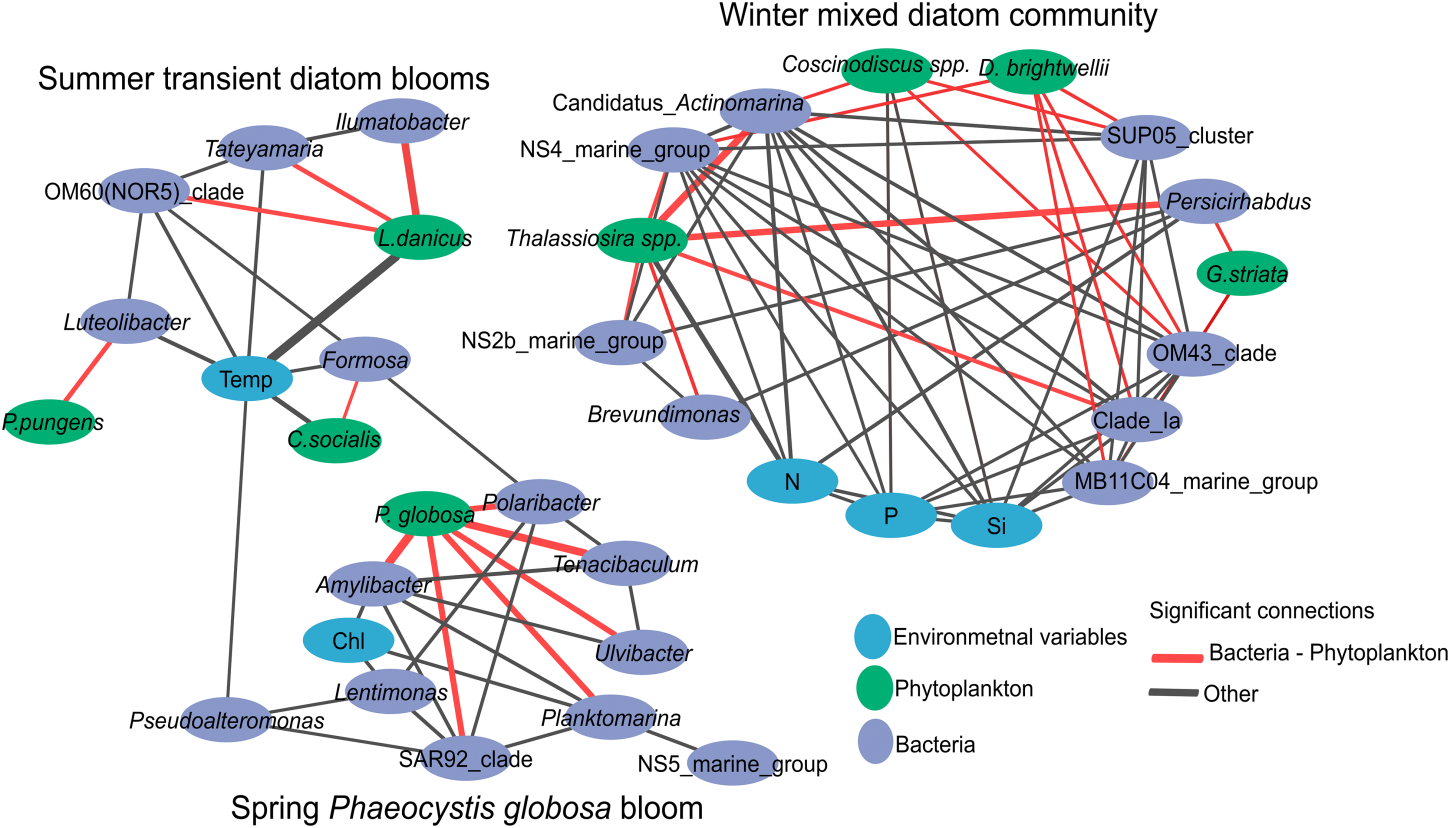
Network diagram of significant correlations (*p* < 0.05) between the 23 dominant bacteria genera (i.e., ≥1% number of reads in 69 samples selected for network analysis), 8 phytoplankton species/genera representing ≥10% of the biomass in 69 samples and 5 environmental variables (Si (OH)_4_, NO_2_ + NO_3_, PO_4_, temperature, and Chl‐*a*) as determined by eLSA analysis without delay. Purple circles represent bacteria, blue ones' environmental variables, and green ones' phytoplankton taxa to facilitate reading. The width of the lines (edges) is proportional to the strength of the association (LS score). Note that all significant connections were positive.

A total of 69 samples were selected for network analysis. Thirty‐eight samples corresponded to dates when blooms occurred in spring (*P. globosa*) and summer (*L. danicus*, *P. pungens*, and *C. socialis*) and 31 winter samples (from December to February) corresponding to a ‘reference’ non‐blooming period (winter) with mixed diatom communities and relatively low bacterial abundance (Figure 2A). In these 69 samples, seven phytoplankton species contributing ≥10% in total phytoplankton biomass (i.e., *Ditylum brightwellii*, *Guinardia striata*, *Chaetoceros socialis, Leptocylindrus danicus, Pseudo‐nitzschia pungens*, and two genera *Thalassiosira* spp., and *Coscinodiscus* spp.), and 28 bacteria genera contributing ≥1% in relative bacterial abundance and environmental variables (temperature, nutrients, salinity and Chl‐*a*) were included in the network analysis. The lag‐delayed and no‐lag networks had similar topological characteristics (Table S8). They identified three distinct subnetworks, corresponding to: (i) the winter ‘non‐blooming’ period characterized by a mixed diatom community, (ii) the ‘*Phaeocystis globosa*’ recurrent spring bloom, and (iii) the summer monospecific ‘diatom blooms.’ The no‐lag subnetworks were better defined (Figure 6 vs. S6), and therefore they will be further discussed here. A comparison of the mean ranks (Kruskal Wallis) of the three subnetworks showed significant differences (i.e., *p* < 0.05) in Characteristic Path Length, Clustering Coefficient, and Edges (Table S9).

Bacterial associations with phytoplankton and environmental variables comprised a total of 36 nodes and 95 edges. The local similarity score (LS) expressed the strength of connection between nodes. The lowest LS score was detected between *Coscinodiscus* spp. and phosphates (i.e., 0.02) and the highest between *L. danicus* and temperature (i.e., 225). The highest number of connections (i.e., 11 edges) was found for Candidatus *Actinomarina* and NS4 marine group (Figure 6).

The ‘winter’ sub‐network had the highest clustering coefficient (mean ± SD, 0.66 ± 0.24) compared to the other two sub‐networks (0.43 ± 0.25, 0.53 ± 0.30; Table S9). In this sub‐network, bacteria were highly interconnected, and they shared connections with phytoplankton and nutrients (Figure 6). The sub‐network consisted of four diatoms (i.e., *Coscinodiscus* spp., *D. brightwellii, G. striata, Thalassiosira* spp.*)*, nine bacteria (i.e., Candidatus *Actinomarina*, SUP05 cluster, *Persicirhabdus*, OM43 clade, Clade Ia, MB11C04 marine group, *Brevundimonas*, NS2b marine group, NS4 marine group) and environmental variables (nitrites and nitrates, phosphates, and silicates) (Figure 6). The most connected bacteria Candidatus *Actinomarina* (i.e., 11 edges) and NS4 marine group (i.e., 11 edges) shared more connections with bacteria than with phytoplankton (i.e., 2 edges). The less‐connected bacterium *Bervudimonas* was connected to two bacteria (NS2b marine group and *Persicirhabdus*). The most connected phytoplankton was *Thalassiosira* spp. (i.e., 7 edges; six connections with bacteria and one with nutrients), followed by *Coscinodiscus* spp. (i.e., 5 edges; three connections with bacteria and two with nutrients), and *D. brightwellii* (i.e., 5 edges; connections with bacteria), while *G. striata* was the less connected one (i.e., 2 edges; connections with bacteria). Out of nine bacteria that consisted of this sub‐network, only three (i.e., MB11C04 marine group, *Brevundimonas*, NS2 marine group) showed unique connections with phytoplankton, while the rest of them were connected with more than one phytoplankton (Figure 6). For example, Candidatus *Actinomarina* was connected with *Coscinodiscus* spp. and *Thalassiosira* spp., and OM43 clade was connected with *D. bridhtwelli* and *G. striata* (Figure 6).

The ‘*P. globosa bloom*’ sub‐network consisted by nine bacteria, *P. globosa*, and Chl‐*a*. Six out of the nine bacteria belonging to this sub‐network were directly connected to *P. globosa* (i.e., *Polaribacter*, *Tenacibaculum*, *Ulvibacter*, *Planktomatina*, SAR92 clade, *and Amylibacter*). They were also connected between them and with the other three bacteria (i.e., *Lentimonas, Pseudoalteromonas*, NS5 marine group). Chl‐*a* was connected with *Amylibacter and Lentimonas* (Figure 6). The ‘Transient diatom blooms’ sub‐network consisted of five bacteria, three diatoms that showed transient peaks in summer, and temperature. This sub‐network evidenced unique connections between phytoplankton and bacteria. *L. danicus* was strongly connected to *Ilumatobacter* and, to a lesser degree, to *Tetayamaria* and OM60(NOR5) clade. *C. socialis* was connected to *Formosa*, and *P. pungens* was connected to *Luteolibacter* (Figure 6).

## DISCUSSION

Previous studies investigated bacteria dynamics in temperate coastal regions have mainly focused on spring/summer diatom blooms (e.g., Arandia‐Gorostidi et al., 2022; Bunse & Pinhassi, 2017; Chafee et al., 2018; Teeling et al., 2016). In this study, bacteria and phytoplankton succession and their putative interactions were investigated in a coastal ecosystem characterized by mixed diatom communities in winter, recurrent blooms of *P. globosa* in spring, and transient diatom blooms in summer. The major findings of this work were that bacterial communities were seasonal and/or substrate‐driven and that diatoms and *P. globosa* promoted distinct bacteria communities. Statistical analyses (i.e., db‐RDA and soft clustering) and network analysis evidenced complementary aspects of the seasonal and species‐specific phytoplankton/bacteria associations (Figures 5 and 6).

### Seasonal dynamics of bacteria and phytoplankton communities

Previous studies have already reported seasonality in bacterioplankton community structure, which is considered to remain recurrent year after year (Fuhrman et al., 2015; Gilbert et al., 2012). Three families (i.e., Actinomarinaceae, Flavobacteriaceae, and Rhodobacteriaceae), accounting for a total 41.2% number of reads, exhibited seasonal variations (Figure 4B). This seasonality was further supported by examining the dynamics at the genus level. For example, Candidatus *Actinomarina* (Actinomatinaceae), which thrives in cold and nutrient‐rich waters, displayed a preference for winter‐autumn seasons (Chafee et al., 2018; Hu et al., 2022; López‐Pérez et al., 2020; Table 1). In winter, phytoplankton communities were dominated by diverse diatoms (Figure S4), favoured by high nutrient concentrations and wind‐driven turbulence (e.g., Breton et al., 2021; Schapira et al., 2008). By the end of March, depletion of silicates and abundant nitrates triggered the growth of *P. globosa* (e.g., Breton et al., 2006). Flavobacteriaceae showed peaks during *P. globosa* blooms, while Rhodobacteraceae or other families dominated summer diatom blooms. Members of the Flavobacteriaceae and Rhodobacteraceae families possess a wide range of hydrolytic enzymes and membrane transporters to degrade and assimilate organic matter, mainly sugars, released by phytoplankton (Pinhassi et al., 2004; Riemann et al., 2000; Teeling et al., 2012). These findings align with a recent experimental study that showed strong seasonal differences in bacterial communities characterized by distinct polysaccharide utilization (laminarin, xylan, and chondroitin) in the North Sea (Giljan et al., 2022). Seasonality was also evident in bacterial abundance and diversity indices (Figures 4A and S5). However, compared to phytoplankton, bacteria showed less pronounced seasonality in alpha diversity indices (Figures S4 and S5). This could be attributed to the large species pool (e.g., 9745 ASVs, 394 genera in the entire dataset) and the consistent availability of substrates provided by phytoplankton communities across seasons.

**TABLE 1:** Bacteria showing significant positive correlations in the network analysis (Figure 6) and strategies (trophic and/or metabolic) reported in previous studies.

Genus	Family	Strategy	References
Mixed diatom community			
Candidatus *Actinomarina*	Actinomarinaceae	Oligotroph, preference in cold waters	e.g., Chafee et al., 2018
*NS4 marine group*	Flavobacteriaceae	Copiotroph	e.g., Lemonnier et al., 2020
*NS2b marine group*	Flavobacteriaceae	Copiotroph	
*MB11C04 marine group*	Puniceicoccaceae	Copiotrophs, specialized consumers of sulfated methyl pentoses	e.g., Orellana et al., 2022
*Clade Ia*	SAR11	Oligotroph	e.g., Giebel et al., 2011
*OM43 clade*	Methylophilaceae	Methylotrophs consume algae C1	e.g., Halsey et al., 2012
*Persicirhabdus*	Rubritaleaceae	Copiotrophs consume complex molecules of organic matter	e.g., Buchan et al., 2014
*SUP05 cluster*	Thoglobaceae	Sulfur‐oxidizing bacteria. Found in anoxic and oxygenated waters	e.g., Chun et al., 2021
*P. globosa* bloom			
*Polaribacter*	Flavobacteriaceae	Copiotroph	e.g., Teeling et al., 2016
*Tenacibaculum*	Flavobacteriaceae	Copiotroph	e.g., Teeling et al., 2016
*Ulvibacter*	Flavobacteriaceae	Copiotroph	e.g., Teeling et al., 2016
*Planktomarina*	Rhodobacteraceae	Copiotroph	e.g., Teeling et al., 2016
*NS5 marine group*	Flavobacteriaceae	Copiotroph	e.g., Teeling et al., 2016
*SAR92 clade*	Porticoccaceae	Copiotroph	e.g., Teeling et al., 2016
*Lentimonas*	Puniceicoccaceae	Copiotroph	e.g., Teeling et al., 2016
*Pseudoalteromonas*	Pseudoalteromonadaceae	Algicidal produces extracellular bioactive molecules to kill phytoplankton cells.	e.g., Mayali & Azam, 2004
*Amylibacter*	Rhodobacteraceae	Copiotroph	e.g., Lemonnier et al., 2020
Transient diatom blooms			
*Formosa*	Flavobacteriaceae	Copiotroph	e.g., Teeling et al., 2016
*Luteolibacter*	Rubritaleaceae	Copiotroph	e.g., Newton & Shade, 2016
*OM60(NOR5) clade*	Halieaceae	Copiotroph	e.g., Spring et al., 2015
*Tateyamaria*	Rhodobacteraceae	Copiotroph	e.g., Teeling et al., 2016

Although the db‐RDA clearly showed a seasonal succession of bacterial communities, the overall contribution of environmental variables explained only about 20% of the community structure variability (i.e., constrained variance: 19.8%; Figure 3; Table S4). This result highlights the importance of organic substrate dependence for bacteria. It is, however, worth noting that the significant contribution of nutrients (*p* < 0.001, Table S6) suggests a meaningful association between nutrients and bacteria, as the latter are involved in nutrient remineralization and uptake (e.g., Kirchman, 1994). In the same vein, temperature is known to be a key environmental factor as it impacts the metabolic kinetics of all organisms (e.g., Brown et al., 2004). Soft clustering evidenced that bacterial clusters were partly overlapped (Figure 5A). Furthermore, about half of the abundant bacteria (≥1% relative abundance in the entire dataset) clustered by season (winter, spring, summer; Figure 5B).

### Exploring taxon‐specific phytoplankton‐bacteria relationships

Network analysis of phytoplankton, bacteria, and environmental variables showed three distinct subnetworks corresponding to (i) winter mixed diatom community; (ii) spring *P. globosa* bloom; and (iii) transient diatom blooms (Figure 6). This was in line with the soft clustering of bacteria, suggesting that phytoplankton is a pivotal driver for bacterial dynamics in this system (Figures 5 and 6). Metabarcoding can overestimate or underestimate the proportions of eukaryotic taxa (e.g., Santi et al., 2021); a strength of the present work is that phytoplankton was enumerated by microscopy avoiding metabarcoding biases. Metabarcoding, biases in bacteria are considered less pronounced than in eukaryotes because bacteria have fewer complex genomes (Cooper, 2000). Also, comparisons of the relative abundance of bacterial families using 16S metabarcoding and staining with specific probes (Fluorescence in Situ Hybridization method) have generally shown accordance (e.g., Teeling et al., 2016).

**FIGURE 7:**
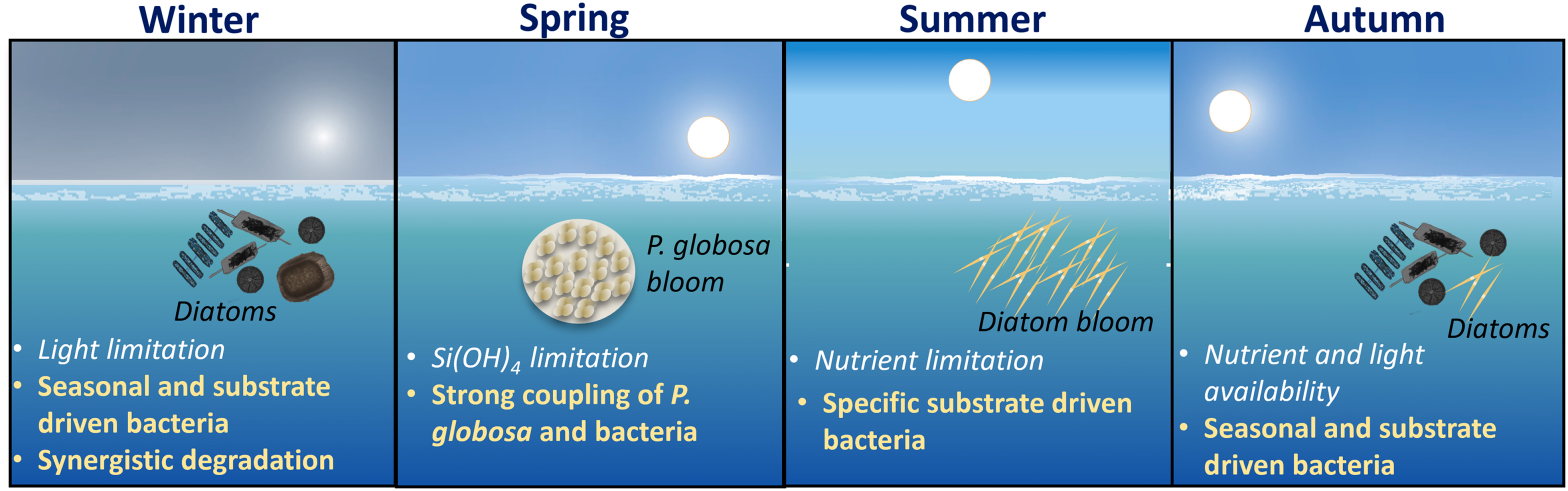
Schematic overview of the seasonal succession and associations between phytoplankton and bacteria in the EEC. In winter, mixed diatom communities were interconnected with bacteria characterized by a wide range of trophic and metabolic strategies (Table 1, Figure 6), indicating a synergistic degradation of diverse phytoplankton‐derived substrates. During recurrent spring blooms, *P. globosa* evidenced strong coupling with bacteria, suggesting a stable‐state environment provided by the mucopolysaccharide matrix of *Phaeocystis* colonies (Figure 6). During transient summer blooms, diatoms showed unique associations with bacteria (Figure 6). Autumn was a transitional period characterized by nutrient and light availability evidenced seasonal and substrate driven bacteria (Figure 6).

The winter sub‐network consisted of well‐connected bacteria between them and with phytoplankton, having various trophic strategies, including copiotrophs and oligotrophs (Table 1, Figure 7). The oligotrophic Candidatus *Actinomarina* dominated in winter and showed various connections with bacteria and diatoms. Candidatus *Actinomarina* is considered a photoheterotroph and found during winter in the North Sea (Chafee et al., 2018) and below the DCM (López‐Pérez et al., 2020). Relatively low‐abundant bacteria (<5% reads in the dataset) also showed several connections. Among these bacteria, the OM43 clade comprises methylotrophs known to feed on algae's C1 compound (i.e., compounds containing one carbon, e.g., methanol; Halsey et al., 2012). They have been associated with phytoplankton spring blooms in the North Sea (Teeling et al., 2016), and they have been found during winter months in the northwestern Atlantic Ocean, suggesting a broad biogeographic distribution at different biotic and abiotic conditions (Georges et al., 2014). Another interesting observation involves the MB11C04 marine group, which was connected to the diatom *D. brightwellii* (Figure 6). This copiotroph bacterium is specialized in consuming complex sulphated methyl pentoses (polysaccharides) produced by diatoms (Orellana et al., 2022). Both OM43 clade and MB11C04 marine group exhibited seasonal patterns (Figure 5). Yet, *Persicirhabdus*, connected to *Thalassiosira* spp., showed variations across years (Figure 5). It is suggested that the type of phytoplanktonic organic matter present in the environment is determinant for *Persicirhabdus* development (Zhang et al., 2015). The intensity of the response of *Persicirhabdus* seems proportional to the substrate quantity (Hellweger, 2018; Lemonnier et al., 2020). This is consistent with our observation of *Thalassiosira* spp. showing variable abundance in winter (0.08–711.4 × 10^3^ cell L^−1^; Figure 2A). The SUP05 cluster, which showed a recurrent seasonal pattern (Figure 5B), has been reported to be abundant at the bottom of the water column and surface when the water column is mixed (Chun et al., 2021). It can be hypothesized that there was a greater variety of substrates derived from a diversified diatom community in winter (Figure S4). Therefore, several bacteria may collaborate to assimilate these substrates. This is consistent with previous studies stressing that carbon is more recalcitrant in winter. Thus, there is a need for various transporters and carbohydrate genes to process it, whereas in summer, carbon is more labile (Ward et al., 2017). Consequently, diverse diatom‐derived substrates required a consortium of interconnected bacteria with specific strategies, including the degradation of simple and complex saccharides.

The ‘*P. globosa*’ sub‐network showed ‘strong’ connections (LS values varied from 108 to 240, which exceeded LS_median_) between *P. globosa* and six copiotroph bacteria (Figure 6; Table 1). Despite the observed variations in the intensity of *P. globosa* blooms over the years (Figure 2B), these six bacteria exhibited recurrent patterns (Figure 5). In a recent study focused on the microbiome of *P. globosa* strains isolated from various geographic regions harboured consistent bacterial communities (Mars Brisbin et al., 2022). *P. globosa*'s microbiome was affiliated with the orders Alteromonadales, Burkholderiales, and Rhizobiales, which promoted the growth of *Phaeocystis* through opportunistic and symbiotic strategies. However, in our study, dominant orders during *P. globosa* bloom was affiliated to Flavobacteriales, Rhodobacteriales, Cellvibrionales, and Opitutales. This discrepancy can be attributed to the fact that while Mars Brisbin et al. (2022) focussed on *P. globosa* colony‐attached bacteria, in our study the protocol for collecting bacteria could not discriminate between free‐living and particle‐attached bacteria. Most likely most of the bacteria found during phytoplankton blooms are free‐living rather than attached to phytoplankton or particles (Teeling et al., 2016). Yet, no definitive conclusions can be drawn regarding this matter which could be the subject of a future study (i.e., differentiate free and attached bacteria during *P. globosa* blooms). The ‘strong’ connections of the six bacteria genera with *P. globosa* (Figure 6) and the recurrence of bacterial composition (at Family and genus level; Figures 2B, 5) during the blooms suggested that *P. globosa* blooms were stable‐state systems for bacteria communities, provided by the unique microenvironment of the mucopolysaccharide matrix (Figure 7). To note, the copiotroph *Pseudoalteromonas* present in the *P. globosa* sub‐network (Figure 6) can be algicidal as it produces extracellular bioactive molecules to kill phytoplankton such as cyanobacteria, dinoflagellates and diatoms (Mayali & Azam, 2004; Seymour et al., 2009). However, to evaluate any algicidal potential on *P. globosa* or other phytoplankton, experimental approaches using algicidal assays are needed (Coyne et al., 2022). The blooming diatoms present in the summer sub‐network showed unique connections with several bacteria (Figure 6). This can be attributed to the different substrates produced by different diatoms (Grossart et al., 2005; Landa et al., 2016). Although previous experimental studies have shown that bacterial communities vary with diatom species (e.g., Landa et al., 2016; Schäfer et al., 2002), this study provides rare in situ evidence of specific associations with diatoms across multiple years.

Finally, co‐occurrence networks should not be over‐interpreted, as they rely on pairwise comparisons (e.g., Blanchet et al., 2020). In that sense, direct connections do not necessarily reveal ‘cause‐effect’ interactions, while the presence of two nodes in the same sub‐network suggests that they co‐vary in similar conditions. According to Hevey (2018) the resulting correlations besides indicating direct relationships, they can also suggest mediation pathways. For example, in the spring sub‐network *P. globosa* was connected to Chl‐*a* through *Planktomarina*, while the diatoms *D. brightwellii* and *G. striata* were associated with silicates through MB11C04 marine group; and *Thalassiosira* spp. was associated to silicates through Ca. *Actinomarina*. To complement observations of the network analysis, statistical analysis db‐RDA and soft‐clustering were realized, and they supported the results of the network analysis (Figures 3 and 5).

Concluding, this study expands previous reports describing the seasonal dynamics of bacterial communities focusing on coastal ecosystems subjected to phytoplankton blooms. Bacterial communities were seasonal and/or substrate‐driven. During winter, in the presence of a diverse diatom community, bacteria were associated with, and between, all phytoplankton species. In contrast, during monospecific *P. globosa* and diatom blooms, specific bacterial genera were strongly associated with the blooming species. In the context of global change, recurrent spring and transient summer blooms are being influenced in terms of length and intensity. Further studies are now needed to explore the functional potential of associated bacterial communities and the importance of algicidal bacteria in terminating *P. globosa* and diatom blooms.

## AUTHOR CONTRIBUTIONS

**Dimitra‐Ioli Skouroliakou:** Investigation; writing – original draft; methodology; validation; visualization; writing – review and editing; software; formal analysis; data curation. **Elsa Breton:** Data curation; methodology; validation. **Urania Christaki:** Conceptualization; investigation; funding acquisition; methodology; validation; writing – review and editing; project administration; data curation; supervision; resources.

## CONFLICT OF INTEREST STATEMENT

The authors declare no conflicts of interest.

## Supporting information


**Appendix S1.** Supplementary information.

## Data Availability

The data that support the findings of this study are available on request from the corresponding author.
