# Supplementary material for: Phaeocystis globosa and diatom blooms promote distinct bacterial communities and associations in a coastal ecosystem
Source: Environ Microbiol Rep. 2024 Jul 10;16(4):e13313. doi: 10.1111/1758-2229.13313 (PMC11236930; doi:10.1111/1758-2229.13313)
Supplement: Supplementary file 1 — Appendix S1. Supplementary information. [file EMI4-16-e13313-s001.docx]

**Supporting Information S1**

**Supporting Materials and Methods**

*DNA barcoding*

The library preparation was performed in a laminar flow hood, and all materials were exposed to UV light for 30 minutes before use to eliminate contamination. The PCR decontamination kit (OZYME, France) removed contaminating DNA from the PCR master mix. All PCRs were performed on four replicates before being pooled together. PCR products were purified using the QIAquick kit, and then the DNA quantity was measured using the Qubit dsDNA HS assay kit.

Different molecular barcodes of 10 bp were added to both forward and reverse primers to tag amplicons and allow to differentiate them after sequencing. Polymerase chain reaction (PCR) mixtures comprised 1 μL of DNA, 12.5 μL of DreamTaq Green PCR Master Mix (Thermo Fisher Scientific, U.S.A.), 1 μL of each primer (10 μmol L^−1^), and 9.5 μL nuclease-free water in a total volume of 25 μL. PCR settings included an initial step of denaturation at 94 ^o^C for 2 min, 25 cycles of denaturation at 94 ^o^C for 15 s, annealing at 58 ^o^C for 30 s, an extension at 72 ^o^C for 1 min and 30 sec, and a final step of extension at 72 ^o^C for 5 min. About 4 μL PCR product was used to check amplification on 1% agarose gel. The remaining amplicon products from five different PCR reactions of each sample were pooled and purified together using the QIAquick PCR purification kit (Qiagen, Germany), according to the manufacturer’s instructions. DNA concentrations after purification were measured with a Qubit 2.0 fluorometer (Thermo Fischer Scientific Inc) with the dsDNA High Sensitivity Assay Kit (Life Technologies Corp., U.S.A.) and adjusted at equal concentrations depending on the sequencing run (22.6 to 47.0 ng/μL).

*Statistical analyses*

Distance-based redundancy analysis (db-RDA) was applied based on Bray-Curtis dissimilarity distance matrix using the ‘microeco’ R package. The significance of db-RDA models was tested with anova permutation test using the *permutest* function with 999 permutations. Soft clustering approach allows assignment of the data points to multiple clusters with varying degrees of membership values. The membership values represent the degree to which a data point belongs to a particular cluster. They are assigned to each data point for each cluster, indicating the strength of the association between that data point and the cluster, and vary between 0 and 1 (i.e., 0 indicates no membership, and 1 indicates full membership; Kauffman and Rousseeuw, 1990). The membership exponent, known as ‘fuzzifier’, was set to 1.1, which determines the cluster's degree of fuzziness (Kauffman and Rousseeuw, 1990).

*Network Analysis*

Local similarity score represents the strength of the correlation between two nodes, and its range depends on the dataset (e.g., Xia *et al.*, 2011; Arandia-Gorostidi *et al.*, 2022). In our dataset, we compared the strength of the associations relative to the mean LS value of the entire dataset (i.e., LS_median_ = 31.5; LS_observed_ ≥ LS_median_ defined as ‘strong’, LS_observed_ < LS_median_, defined as ‘weak’). Weiss et al., (2016) compared eLSA with seven other network analysis methods (e.g., SparCC, MIC, CoNet) and found that eLSA was the most suitable for time-series data. They also highlighted that eLSA is capable of correctly inferring three-member relationships. The number of positive and negative edges and connected nodes characterize a network. Regarding the network characteristics included in this study; the network *mean degree* corresponds to the average number of edges each node establishes. The *Characteristic Path Length* represents the average shortest number of steps (number of nodes) between all node pairs. The *Clustering Coefficient represents* the network complexity, which is the probability of two nodes having a similar neighbor being connected (Delmas et al., 2019). The *Clustering Coefficient* varies between 0 and 1; low values indicate globally poorly connected neighborhoods.

**S2. Supporting Figures**


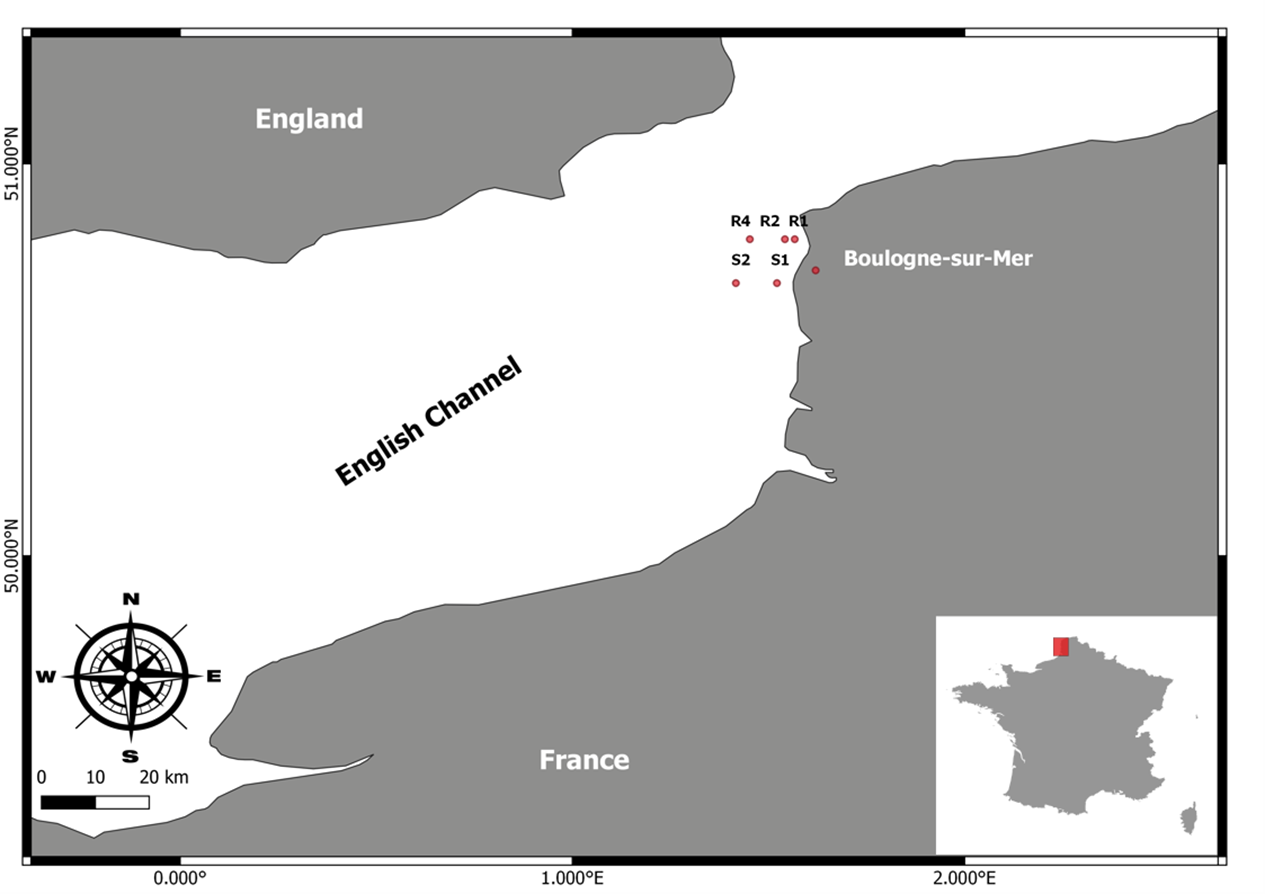


**Figure S1**. Location of the SOMLIT (S1, S2) and DYPHYRAD (R1, R2, R4) stations in the EEC (map creation with QGIS software V.3.10.1).

***
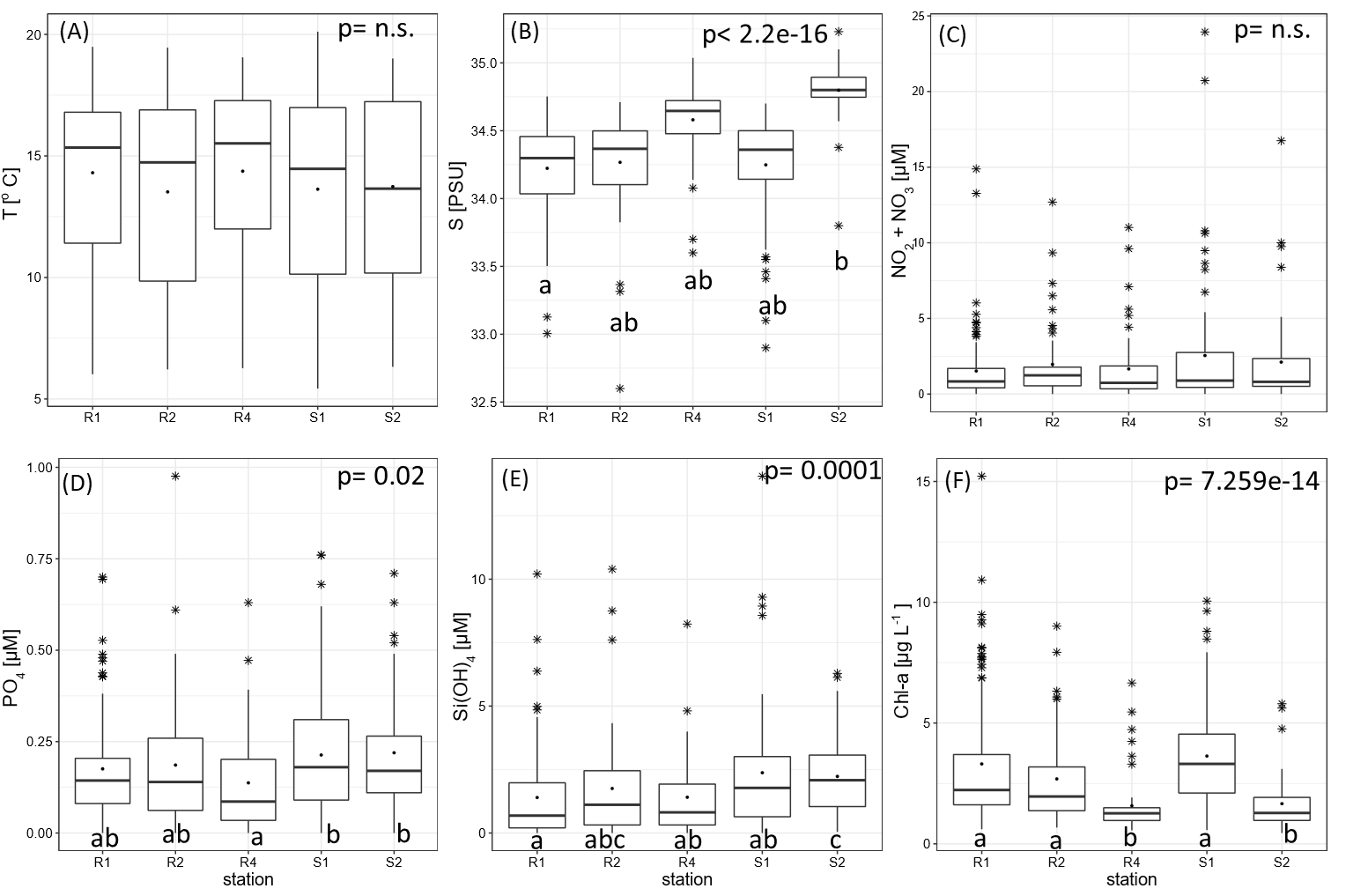
***

**Figure S2**. Spatial variation of environmental variables measured in the EEC at the DYPHYRAD and SOMLIT stations from March 2016 to October 2020: (A) Temperature [T, ^o^C], (B) S: Salinity [S, SSU], (C) Nitrites and Nitrates [NO_2_+NO_3_ μM], (D) Phosphates [PO_4_, μM], (E) Silicates: [Si (OH)_4_, μM], (F) Chlorophyll-a [Chl-*a*, μg L^-1^]. The letters indicate significant differences (p < 0.05) between stations based on the Kruskal-Wallis and Nemenyi post-hoc test on the boxplot's top and bottom, respectively. Solid black lines represent the median, black dots represent the mean, and black stars represent the outliers.

***
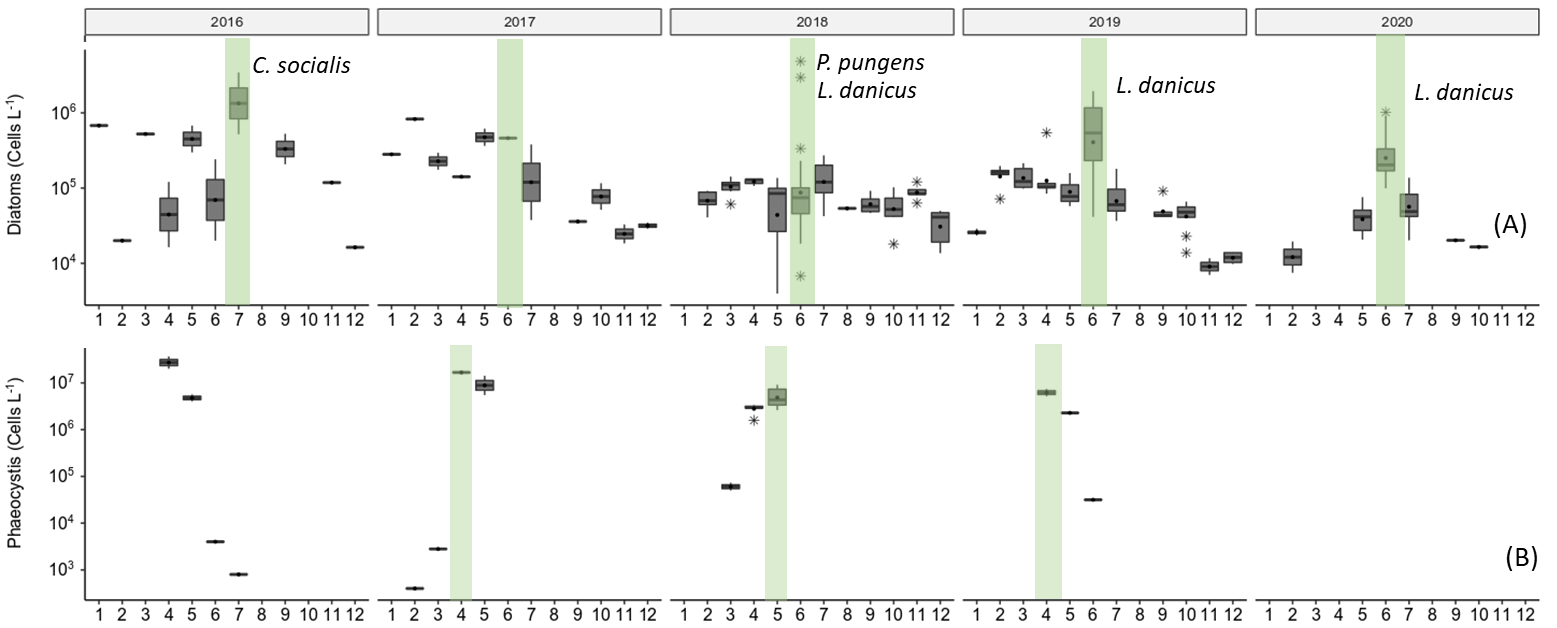
***

**Figure S3**. Abundance (cells L^-1^) of phytoplankton (diatoms and *Phaeocystis globosa*) identified by microscopy in the EEC at the SOMLIT and DYPHYRAD stations from March 2016 to October 2020 based on microscopy and flow cytometry data., (A) diatoms, (B) *Phaeocystis globosa*. The y-axis is log10 transformed. No data were available from February 14, 2020, to May 20, 2020, because of the sanitary crisis and on August due to the annual leave of the personnel). Solid black lines represent the median, black dots the mean, and the black stars the outliers. The green stripe indicates the period presenting peaks.


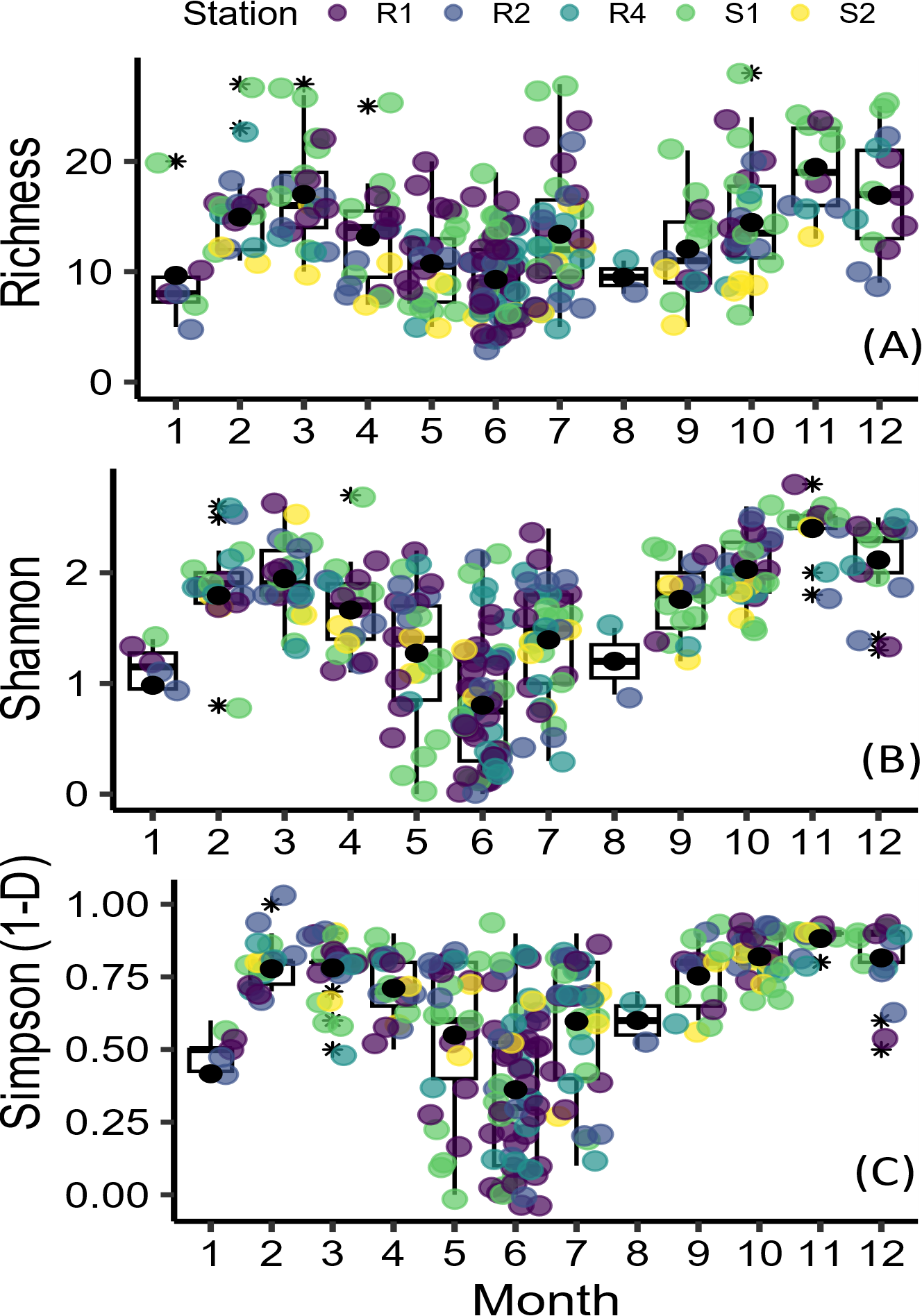


**Figure S4**. Alpha diversity variations of diatom communities in the EEC at the DYPHYRAD and SOMLIT stations from March 2016 to October 2020. (A) Richness, (B) Shannon and (C) Simpson (1-D) indices. Solid black lines represent the median, black dots represent the mean, colored dots represent the samples according to stations and years, and black stars represent the outliers.

***
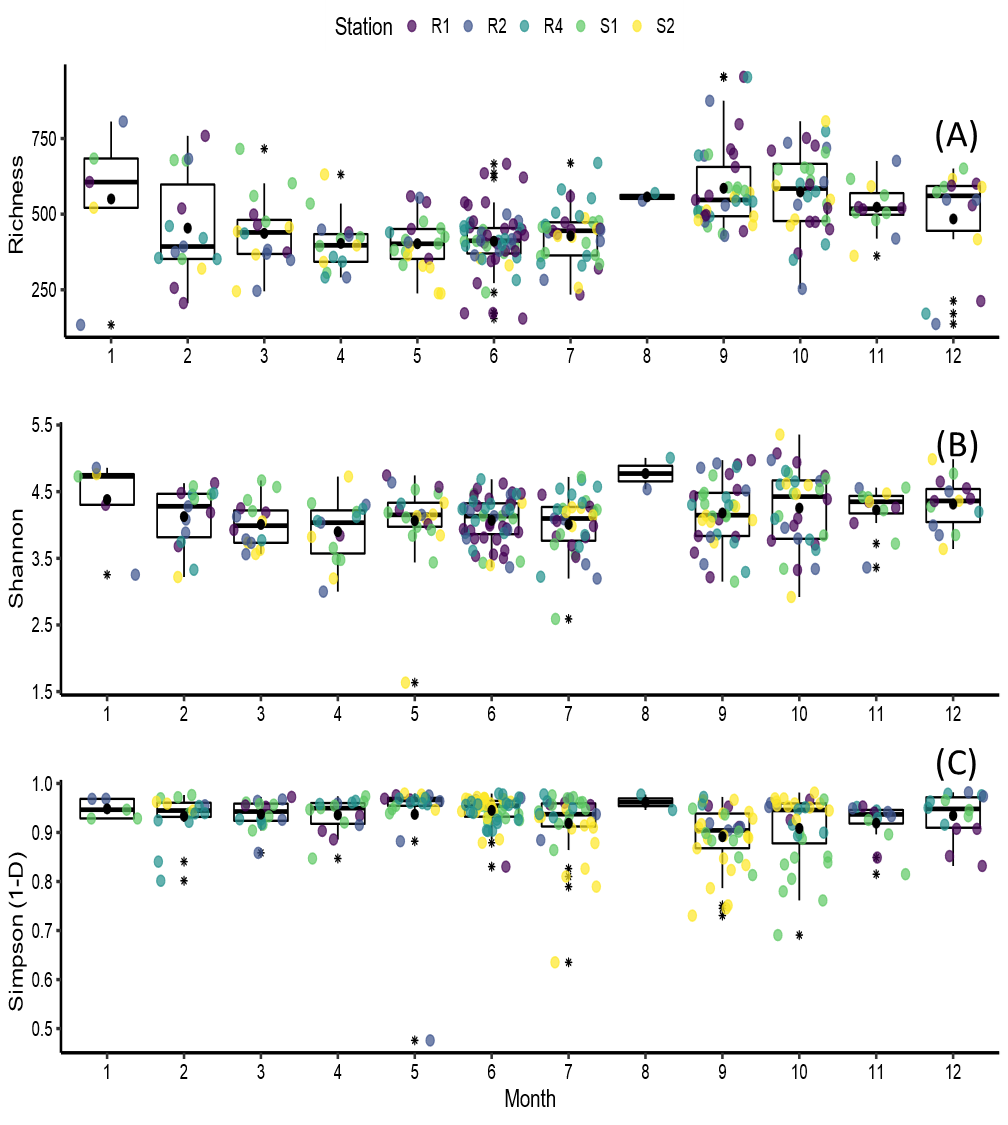
***

**Figure S5**. Alpha diversity variations of the heterotrophic bacterial communities in the EEC at the DYPHYRAD and SOMLIT stations from March 2016 to October 2020. (A) Richness, (B) Shannon and (C) Simpson (1-D) indices. All samples were rarefied at 8,000 reads. Solid black lines represent the median, black dots represent the mean, colored dots represent the samples according to stations and years, and black stars represent the outliers.

***
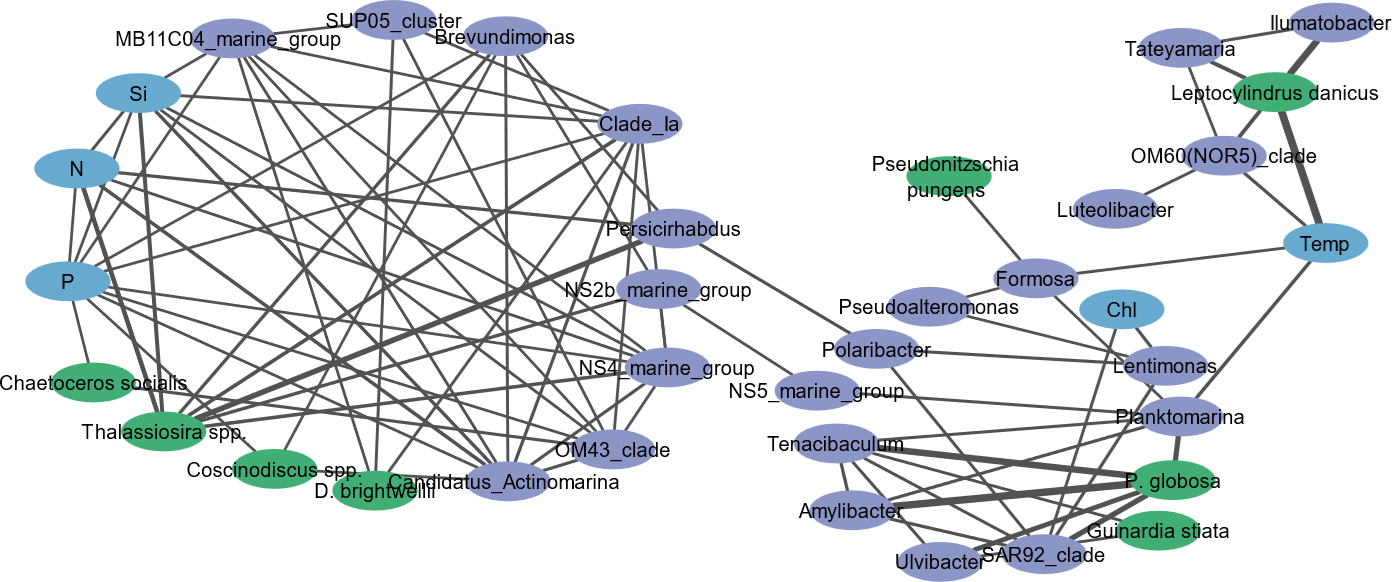
***

**Figure S6**. Network diagram of positively significant correlations (p<0.05) between the 22 dominant bacteria, 8 phytoplankton taxa (see Material and Methods), 5 environmental variables (Si(OH)_4_, NO_2_+NO_3_, PO_4_, temperature, and Chl-*a*) as determined by eLSA analysis using a delay of one-time point. To facilitate this, purple circles represent the bacteria, blue ones represent the environmental variables, and green ones represent the phytoplankton taxa. The width of the lines (edges) corresponds to the LS score.

**Supporting tables**

**Table S1**. Station and sample description. Max. Depth corresponds to the highest tide. Note that S1 and R1, being closer to the coast, were easier to sample under difficult weather conditions (see also Fig. S1). Environmental parameters and 16S rRNA gene amplicon sequencing were realized for all samples (i.e., 282) from 2016 to 2020. Microscopic counts for phytoplankton and flow cytometry were acquired from 2018 to 2020 at all stations.

| Station | Date | Long (°E) | Lat (°N) | Max. depth | Distance from the coast (km) | Sampling  Frequency | No of samples |
| --- | --- | --- | --- | --- | --- | --- | --- |
| S1 | 2016-2020 | 1.3117 | 50.4075 | 27 | 2 | Bi-weekly | 65 |
| S2 | 2016-2020 | 1.2460 | 50.4075 | 56 | 9.8 | Bi-weekly | 41 |
| R1 | 2018-2020 | 1.3360 | 50.4760 | 19 | 2.6 | Once/twice a week | 85 |
| R2 | 2018-2020 | 1.3231 | 50.4760 | 23 | 4.3 | Once a week | 43 |
| R4 | 2018-2020 | 1.2780 | 50.4760 | 52 | 10.9 | Once/twice a week | 48 |

**Table S2**. Cell-to-carbon biomass conversion factors were used in this study to estimate the biomass of *Phaeocystis globosa* and diatoms identified in the EEC at the SOMLIT and DYPHYRAD stations from March 2016 to October 2020.

|  | Conversion factor | References |
| --- | --- | --- |
| Diatoms | pgC cell^-1^=0.288 x (biovolume)^0.811^ | Based on linear dimensions, the biovolume was calculated according to the cell shape (Hillebrand *et al*., 1999). Conversion from biovolume to biomass, according to Menden Deuer & Lessard 2000 and based on microscopic observations |
| *Phaeocysti*s free flagellate cells | 8 pgC cell^-1^ | Schoeman *et al*., 2005 |
| *Phaeocystis* colonial cells | 14.2 pgC cell^-1^ |  |

**Table S3** Monthly range, mean (±SD), and median values of the mean sea surface temperature (T, °C), salinity (S, PSU), nutrients (nitrite and nitrate: NO_2_ + NO_3_, phosphate: PO_4_, all in μM, the Ν/P molar ratio, silicate Si(OH)_4_, μΜ, chlorophyll-a (Chl-*a*, μg L^-1^) in the EEC at the DYPHYRAD and SOMLIT stations from March 2016 to October 2020. Note that only three samples were available for August.

|  | January | February | March | April | May | June | July | August | September | October | November | December |
| --- | --- | --- | --- | --- | --- | --- | --- | --- | --- | --- | --- | --- |
| T  (°C) |  |  |  |  |  |  |  |  |  |  |  |  |
| Range | 5.4-7.9 | 6.0-8.3 | 5.8-9.5 | 8.6-11.4 | 11.03-14.7 | 13.7-18.2 | 16.5-20 | 19.1-19 | 12.4-19.8 | 14.4-18 | 10.6-14.9 | 8.1-11.4 |
| Mean±SD | 7.1±0.8 | 7.4±0.7 | 8.0±1.3 | 9.6±0.7 | 12.91±1.06 | 15.4±1.0 | 17.8±0.8 | 19.3±0.2 | 18.1±1.3 | 16.2±0.9 | 12.6±1.3 | 10.2±0.8 |
| Median | 7 | 7.5 | 8.2 | 9.7 | 12.86 | 15.4 | 17.8 | 19.5 | 18.6 | 16.4 | 12.8 | 10.4 |
| CV(%) | 11.8 | 10.2 | 16.6 | 7.5 | 8.19 | 6.4 | 4.2 | 1.2 | 7.4 | 5.4 | 10.4 | 7.9 |
| S  (PSU) |  |  |  |  |  |  |  |  |  |  |  |  |
| Range | 33.8-34.9 | 32.9-34.8 | 33.3-34.9 | 32.6-35.2 | 33.5-35.0 | 31.2-35.1 | 33.4-34.9 | 0.03-0.1 | 33.8-34.9 | 33.6-35 | 34.1-35.0 | 33.9-35.0 |
| Mean±SD | 34.2±0.4 | 34.0±0.6 | 34.2±0.5 | 34.1±0.6 | 34.2±0.4 | 34.3±0.5 | 34.6±0.3 | 34.6±0.2 | 34.5±0.2 | 34.5±0.3 | 34.6±0.3 | 34.4±0.3 |
| Median | 34.1 | 34.1 | 34.2 | 34.2 | 34.09 | 34.4 | 34.6 | 34.6 | 34.6 | 34.5 | 34.6 | 34.3 |
| CV(%) | 1 | 1.7 | 1.5 | 1.7 | 1.27 | 1.4 | 0.9 | 0.5 | 0.5 | 0.8 | 0.8 | 0.8 |
| NO_2_+NO_3_ (µM) |  |  |  |  |  |  |  |  |  |  |  |  |
| Range | 3.4-10.6 | 0.3-23.9 | 1.1-20.7 | 0.3-9.3 | 0.14-1.6 | 0.01-4.4 | 0.2-1.1 | 0.2-0.5 | 0.3-2.8 | 0.2-3.5 | 0.4-5.2 | 1.1-8.6 |
| Mean±SD | 8.0±4.0 | 7.6±6.6 | 5.2±4.4 | 2.2±2.3 | 0.53±0.4 | 0.9±1.0 | 0.5±0.2 | 0.4±0.2 | 1.0±0.6 | 1.5±0.8 | 2.2±1.4 | 3.5±2.4 |
| Median | 10 | 4.2 | 4.2 | 1.5 | 0.41 | 0.5 | 0.5 | 0.5 | 0.7 | 1.5 | 1.8 | 2.9 |
| CV(%) | 49.8 | 86.5 | 85.2 | 104.1 | 74.01 | 113.5 | 42 | 44.2 | 67.8 | 52.4 | 66.3 | 68.6 |
| PO_4_ (µM) |  |  |  |  |  |  |  |  |  |  |  |  |
| Range | 0.4-0.6 | 0.2-1.0 | 0.01-0.6 | 0.03-0.3 | 0.01-0.29 | 0.01-0.4 | 0.01-0.2 | 0.1-0.2 | 0.01-0.5 | 0.03-0.4 | 0.01-0.5 | 0.1-0.8 |
| Mean±SD | 0.5±0.1 | 0.5±0.2 | 0.3±0.1 | 0.1±0.1 | 0.1±0.07 | 0.1±0.1 | 0.1±0.05 | 0.1±0.1 | 0.17±0.1 | 0.2±0.1 | 0.3±0.2 | 0.4±0.2 |
| Median | 0.5 | 0.5 | 0.2 | 0.2 | 0.11 | 0.1 | 0.1 | 0.1 | 0.14 | 0.2 | 0.2 | 0.4 |
| CV(%) | 14 | 39.3 | 51.3 | 47 | 71.24 | 91.7 | 57 | 52.2 | 64 | 46.5 | 56.4 | 47.3 |
| N/P |  |  |  |  |  |  |  |  |  |  |  |  |
| Range | 7.0-24.2 | 0.4-35.2 | 4.8-52.7 | 1.5-43.7 | 0.82-262.5 | 2.1-316.1 | 1.1-15.3 | 3.0-3.9 | 2.3-78.0 | 2.7-16.3 | 0.4-5.2 | 3.8-21.1 |
| Mean±SD | 16.6±8.7 | 14.7±10.5 | 18.5±13.2 | 13.0±12.0 | 25.71±61.0 | 17.4±42.4 | 7.3±3.3 | 3.4±0.4 | 8.4±12.2 | 7.7±2.7 | 2.2±1.4 | 9.1±4.6 |
| Median | 18.5 | 9.4 | 14.6 | 9.3 | 4.22 | 7.5 | 6.5 | 3.3 | 5.3 | 7.1 | 1.8 | 9 |
| CV(%) | 52.8 | 71.8 | 71.7 | 92 | 237.3 | 243.6 | 44.8 | 12.2 | 145 | 35 | 66.3 | 50.2 |
| Si(OH)_4_ (µM) |  |  |  |  |  |  |  |  |  |  |  |  |
| Range | 4.6-8.8 | 1.8-14.1 | 0.1-5.2 | 0.1-3.6 | 0.05-2.68 | 0.01-3.0 | 0.01-3.4 | 1.1-2.9 | 0.01-4.8 | 0.1-6.1 | 0.3-5.5 | 1.0-9.3 |
| Mean±SD | 5.9±1.9 | 5.2±3.6 | 1.9±1.5 | 0.9±0.8 | 1.04±0.70 | 0.9±0.9 | 1.3±1.1 | 2.1±0.9 | 1.2±1.1 | 1.8±1.7 | 2.7±1.5 | 4.6±2.7 |
| Median | 5.1 | 3.3 | 1.8 | 0.6 | 0.9 | 0.6 | 1.2 | 2.3 | 0.7 | 1.5 | 3.1 | 4.3 |
| CV(%) | 33 | 68.3 | 79.2 | 88.9 | 67.52 | 96.5 | 84.2 | 41.9 | 92.8 | 92.7 | 53.5 | 58.6 |
| Chl-*a*  (µg L-1) |  |  |  |  |  |  |  |  |  |  |  |  |
| Range | 1.01- 2.7 | 0.6-7.9 | 1.6-9.3 | 0.6-15.2 | 0.6-10.9 | 0.6-9.6 | 0.6-8.5 | 0.8-1.5 | 0.5-7.9 | 1.0-6.9 | 0.7-4.6 | 0.5-3.5 |
| Mean±SD | 1.7±0.6 | 3.9±2.2 | 5.1±2.3 | 5.7±3.1 | 2.9±3.0 | 2.3±1.7 | 2.1-1.5 | 1.1±0.3 | 1.6±1.2 | 2.4±1.3 | 2.5±1.4 | 1.5±0.9 |
| Median | 1.6 | 3.5 | 5.1 | 5.4 | 1.7 | 1.8 | 1.7 | 0.9 | 1.3 | 2 | 2.6 | 1.3 |
| CV(%) | 33.7 | 56.7 | 44.3 | 54.9 | 102.2 | 74.1 | 70.8 | 32.6 | 73.5 | 53 | 56.9 | 61.2 |

**Table S4.** Partitioning of squared distance of distance-based redundancy analysis (db-RDA). The constrained variance (i.e., 19.8 %) is highlighted in bold.

|  | Inertia | Proportion |
| --- | --- | --- |
| Total | 56.51 | 1 |
| Constrained | 11.16 | **0.1976** |
| Unconstrained | 45.35 | 0.8024 |

**Table S5.** Importance of db-RDA components. The proportions explained by db-RDA1 and dbRDA2 are highlighted in bold.

|  | db-RDA1 | db-RDA2 | db-RDA3 | db-RDA4 | db-RDA5 | db-RDA6 |
| --- | --- | --- | --- | --- | --- | --- |
| Eigenvalue | 6.7537 | 2.54144 | 0.67138 | 0.55409 | 0.424 | 0.22021 |
| Proportion Explained | **0.1195** | **0.04497** | 0.01188 | 0.00981 | 0.0075 | 0.0039 |

**Table S6.** Permutation ANOVA (999 permutations) on the final distance-based redundancy model. The three most significant environmental variables are highlighted in bold.

| Environmental variables | Data frame | Sum of squares | F | p |
| --- | --- | --- | --- | --- |
| **Temperature** | **1** | **3.624** | **17.9792** | **0.001** |
| Salinity | 1 | 0.861 | 4.2734 | 0.001 |
| **Nitrites and Nitrates** | **1** | **2.274** | **11.2828** | **0.001** |
| **Phosphates** | **1** | **3.315** | **16.4498** | **0.001** |
| Silicates | 1 | 0.385 | 1.9085 | 0.042 |
| Chlorophyll a | 1 | 0.706 | 3.5008 | 0.003 |

**Table S7**. Membership value of abundant bacteria (contributing in ≥ 1 % relative abundance in the entire dataset) to each cluster based on soft clustering analysis (See Fig. 5A). Membership values greater than 0.7 are indicated in green.

|  | **Cluster 1** | **Cluster 2** | **Cluster 3** | **Cluster 4** | **Cluster 5** |
| --- | --- | --- | --- | --- | --- |
| Candidatus_*Actinomarina* | 1 | 0 | 0 | 0 | 0 |
| *Amylibacter* | 0 | 0.001 | 0.996 | 0 | 0.002 |
| *Persicirhabdus* | 0 | 0.008 | 0.978 | 0.003 | 0.012 |
| *Planktomarina* | 0 | 0 | 0.997 | 0 | 0.002 |
| *Lentimonas* | 0 | 0.005 | 0.975 | 0.002 | 0.018 |
| NS5_marine_group | 0 | 0.006 | 0.977 | 0 | 0.017 |
| *Ilumatobacter* | 0 | 0 | 0 | 1 | 0 |
| *Tenacibaculum* | 0 | 0.003 | 0.975 | 0.001 | 0.021 |
| OM60.NOR5.clade | 0 | 0.015 | 0.007 | 0 | 0.978 |
| Clade_Ia | 0 | 0.998 | 0 | 0 | 0.002 |
| NS4_marine_group | 0 | 0.992 | 0 | 0 | 0.008 |
| *Luteolibacter* | 0 | 0 | 0 | 0.999 | 0 |
| SAR92_clade | 0 | 0.003 | 0.013 | 0 | 0.984 |
| OM43_clade | 0 | 0.999 | 0 | 0 | 0.001 |
| SUP05_cluster | 0 | 0.999 | 0 | 0 | 0.001 |
| MB11C04_marine_group | 0 | 0.996 | 0 | 0 | 0.004 |
| *Polaribacter* | 0 | 0.005 | 0.015 | 0.001 | 0.98 |
| *Roseibacillus* | 0 | 0.026 | 0 | 0 | 0.973 |
| *Pseudohongiella* | 0 | 0.023 | 0 | 0 | 0.977 |
| *Blastopirellula* | 0 | 0.113 | 0 | 0 | 0.886 |
| *Formosa* | 0 | 0.002 | 0 | 0 | 0.998 |

**Table S8**. Network statistics of the eLSA analysis without lag and with lag delay of 1 time point. The connections with p < 0.05 are shown. All topological parameters were calculated with Cytoscape v.3.9.1 (Shannon et al., 2003).

|  | no lag | lag 1 |
| --- | --- | --- |
| Nodes | 36 | 36 |
| Edges (pairs) | 95 | 84 |
| Avg. number of neighbors | 3,900 | 4,667 |
| Network diameter | 6 | 8 |
| Network radius | 3 | 4 |
| Characteristic path length | 2,737 | 3,757 |
| Clustering coefficient | 0.478 | 0.529 |
| Network density | 0.205 | 0.133 |
| Network heterogeneity | 0.421 | 0.539 |
| Network centralization | 0.181 | 0.161 |
| Connected components | 2 | 1 |

**Table S9**. Topological statistics (Characteristic Path Length, Clustering Coefficient, Edges) of the network without lag corresponding to bacteria, phytoplankton, and environmental variables shown in the network (Fig. 6).

| **Sub-networks** | **Characteristic Path Length** | **Clustering Coefficient** | **Edges** |
| --- | --- | --- | --- |
| **Mixed-diatom community (winter)** |  |  |  |
| **Phytoplankton** |  |  |  |
| *Thalassiosira* spp*.* | 1.53 | 0.52 | 7.00 |
| *Coscinodiscus* spp*.* | 1.87 | 0.90 | 5.00 |
| *D. brightwellii* | 1.80 | 1.00 | 5.00 |
| *Guinardia stiata* | 1.93 | 0.00 | 2.00 |
| **Bacteria** |  |  |  |
| Candidatus*_Actinomarina* | 1.27 | 0.58 | 11.00 |
| *NS4_marine_group* | 1.27 | 0.58 | 11.00 |
| *NS2b_marine_group* | 1.67 | 0.60 | 5.00 |
| *SUP05_cluster* | 1.60 | 0.79 | 8.00 |
| *MB11C04_marine_group* | 1.47 | 0.67 | 9.00 |
| *Persicirhabdus* | 1.93 | 0.40 | 5.00 |
| *Clade_Ia* | 1.40 | 0.72 | 9.00 |
| *OM43_clade* | 1.53 | 0.78 | 9.00 |
| *Brevundimonas* | 2.27 | 1.00 | 3.00 |
| **Environmental variables** |  |  |  |
| NO_2_ | 1.60 | 0.60 | 6.00 |
| PO_3_ | 1.53 | 0.75 | 8.00 |
| Si(OH)_4_ | 1.47 | 0.75 | 9.00 |
| **Mean±SD** | **1.63±0.27** | **0.66±0.24** | **7.00±2.68** |
| ***P. globosa* bloom (spring)** |  |  |  |
| **Phytoplankton** |  |  |  |
| *P. globosa* | 2.47 | 0.53 | 6.00 |
| **Bacteria** |  |  |  |
| *Polaribacter* | 2.16 | 0.30 | 5.00 |
| *Tenacibaculum* | 2.68 | 0.67 | 4.00 |
| *Ulvibacter* | 3.26 | 1.00 | 3.00 |
| *Lentimonas* | 2.37 | 0.33 | 4.00 |
| *SAR92_clade* | 2.16 | 0.40 | 6.00 |
| *Planktomarina* | 2.68 | 0.40 | 5.00 |
| *NS5_marine_group* | 3.63 | 0.00 | 1.00 |
| *Amylibacter* | 2.63 | 0.47 | 6.00 |
| *Pseudoalteromonas* | 2.11 | 0.33 | 3.00 |
| **Environmental variables** |  |  |  |
| Chl-*a* | 2.79 | 0.33 | 3.00 |
| **Mean±SD** | **2.63±0.47** | **0.43±0.25** | **4.18±1.60** |
| **Transient diatom blooms**  **(summer)** |  |  |  |
| *Chaetoceros socialis* | 2.79 | 1.00 | 2.00 |
| *Leptocylindrus danicus* | 2.84 | 0.67 | 4.00 |
| *Pseudo-nitzschia pungens* | 3.84 | 0.00 | 1.00 |
| **Bacteria** |  |  |  |
| *Luteolibacter* | 2.89 | 0.33 | 3.00 |
| *Formosa* | 2.21 | 0.33 | 4.00 |
| *Ilumatobacter* | 3.74 | 1.00 | 2.00 |
| *Tateyamaria* | 2.84 | 0.67 | 4.00 |
| *OM60(NOR5) clade* | 2.53 | 0.50 | 5.00 |
| **Environmental variables** |  |  |  |
| Temp | 2.11 | 0.29 | 7.00 |
| **Mean±SD** | **2.87±0.6** | **0.53±0.3** | **3.56±1.8** |

**References**

Arandia-Gorostidi, N., Krabberød, A.K., Logares, R., Deutschmann, I.M., Scharek, R., Morán, X.A.G., et al. (2022) Novel Interactions Between Phytoplankton and Bacteria Shape Microbial Seasonal Dynamics in Coastal Ocean Waters. *Front Mar Sci* **9**: 901201.

Delmas, E., Besson, M., Brice, M.-H., Burkle, L.A., Dalla Riva, G.V., Fortin, M.-J., et al. (2019) Analysing ecological networks of species interactions. *Biol Rev* **94**: 16–36.

Kauffman, L. and Rousseeuw, P.J. (1990) Finding Groups in Data: An introduction to cluster analysis. In *Finding Groups in Data*. John Wiley & Sons, Ltd, pp. 320–331.

Weiss, S., Van Treuren, W., Lozupone, C., Faust, K., Friedman, J., Deng, Y., et al. (2016) Correlation detection strategies in microbial data sets vary widely in sensitivity and precision. *ISME J* **10**: 1669–1681.

Xia, L.C., Steele, J.A., Cram, J.A., Cardon, Z.G., Simmons, S.L., Vallino, J.J., et al. (2011) Extended local similarity analysis (eLSA) of microbial community and other time series data with replicates. *BMC Syst Biol* **5**: S15.
